# Anticipated Guilt for Not Helping and Anticipated Warm Glow for Helping Are Differently Impacted by Personal Responsibility to Help

**DOI:** 10.3389/fpsyg.2016.01475

**Published:** 2016-09-28

**Authors:** Arvid Erlandsson, Amanda Å. Jungstrand, Daniel Västfjäll

**Affiliations:** ^1^Department of Behavioral Sciences and Learning, Linköping UniversityLinköping, Sweden; ^2^Department of Psychology, Lund UniversityLund, Sweden; ^3^Decision ResearchEugene, OR, USA

**Keywords:** anticipated guilt, anticipated warm glow, emotion regulation, motivations of helping, negative state relief model, responsibility to help

## Abstract

One important motivation for people behaving prosocially is that they want to avoid negative and obtain positive emotions. In the prosocial behavior literature however, the motivations to avoid negative emotions (e.g., guilt) and to approach positive emotions (e.g., warm glow) are rarely separated, and sometimes even aggregated into a single mood-management construct. The aim of this study was to investigate whether anticipated guilt if not helping and anticipated warm glow if helping are influenced similarly or differently when varying situational factors related to personal responsibility to help. Helping scenarios were created and pilot tests established that each helping scenario could be formulated both in a high-responsibility version and in a low-responsibility version. In Study 1 participants read high-responsibility and low-responsibility helping scenarios, and rated either their anticipated guilt if not helping or their anticipated warm glow if helping (i.e., separate evaluation). Study 2 was similar but here participants rated both their anticipated guilt if not helping and their anticipated warm glow if helping (i.e., joint evaluation). Anticipated guilt was clearly higher in the high-responsibility versions, but anticipated warm glow was unaffected (in Studies 1a and 1b), or even higher in the low-responsibility versions (Study 2). In Studies 3 (where anticipated guilt and warm glow were evaluated separately) and 4 (where they were evaluated jointly), personal responsibility to help was manipulated within-subjects. Anticipated guilt was again constantly higher in the high-responsibility versions but for many types of responsibility-manipulations, anticipated warm glow was higher in the low-responsibility versions. The results suggest that we anticipate guilt if not fulfilling our responsibility but that we anticipate warm glow primarily when doing over and beyond our responsibility. We argue that future studies investigating motivations for helping should measure both anticipated negative consequences for oneself if not helping, and anticipated positive consequences for oneself if helping.

## Introduction

Imagine that you hear that the blood-supplies are running low in all hospitals in your part of the country. You know that you could help by visiting the nearby donor clinic on your way back from work and donate blood (it would take you around 30 min). Now imagine a similar situation, but where the donor clinic is located far away (it would take you around 4 h to go there and donate blood). How would you feel in both these situations in case you decided to help, or in case you decided not to help?

The current study investigates people's anticipated emotional reactions when they consider helping and non-helping in high- and low-responsibility situations. In the example above, responsibility to help varied as a result of the personal effort it would take to help, but personal responsibility can be varied by manipulating many different types of situational factors. Our central hypothesis is that people anticipate stronger negative feelings (e.g., guilt) if not helping in high-responsibility situations (e.g., not donating blood when the clinic is near), compared to if not helping in low-responsibility situations (e.g., not donating blood when the clinic is far away), but that they anticipate stronger positive feelings (e.g., warm glow) if helping in low-responsibility situations (e.g., donating blood when the clinic is far away) compared to helping in high-responsibility situations (e.g., donating blood when the clinic is near).

Our research question is embedded primarily in the literature about underlying emotional mechanisms of prosocial behavior. The key question to be addressed here is whether or not anticipated guilt if not helping and anticipated warm glow if helping should be considered two separate constructs or two parts of a single mood-improvement construct. This research is important because much previous research on underlying motives for prosocial behavior has either, implicitly or explicitly, treated guilt-avoidance and warm glow-attainment as two sides of the same coin (e.g., Cialdini et al., 1987; Dickert et al., 2011; Ferguson et al., 2012), or solely focused on the determinants of one of these anticipated emotions (e.g., Lindsey, 2005; Cryder et al., 2013). Establishing whether anticipated guilt and anticipated warm glow are differently determined is important given that many theories of prosocial giving assign a primary role for both negative and positive emotions in motivating action (e.g., Slovic, 2007).

To help guide our studies, we first review research on the emotional underpinnings of helping. We first focus on the negative emotion of guilt and specifically on how anticipated guilt influences helping. We then note that there is a difference between helping in order to avoid emotional punishment (i.e., guilt if not helping), and helping in order to approach emotional reward (i.e., warm glow if helping), and discuss the literature on anticipated warm glow. The second part reviews research related to the concept of responsibility to help. There, we first discuss how perceived responsibility can increase helping, and then provide a rationale for our hypothesis that anticipated guilt if not helping and anticipated warm glow if helping will be differently influenced by varying situational aspects related to personal responsibility in helping situations.

## Emotional underpinnings of helping

It is widely acknowledged that affect (e.g., Slovic, 2007) and emotions (e.g., Haidt, 2003) are crucial in helping decisions. A need-situation that does not give rise to any emotions will likely not render any helping behavior. Many types of emotional reactions have been suggested as possible reasons for helping, but the current study will focus primarily on guilt and warm glow.

### Guilt, anticipated guilt, and helping

Guilt is an emotion that has received much attention (see Baumeister et al., 1994; Tangney and Tracy, 2012), and its relation to helping has been established many times (Hibbert et al., 2007; Miller, 2010; Ahn et al., 2014). Guilt is often defined as a negative, self-directed emotional reaction that arises as a result of behaving immorally. Although guilt is often stronger after doing something bad (e.g., harming), a central assumption in this paper is that guilt can also follow from not doing something good (e.g., not helping; O'Keefe and Figgé, 1999; Lindsey, 2005). Importantly, guilt can refer to different things and influence helping at different stages of the decision process (Västfjäll et al., 2016). Both experienced *integral* guilt (a negative emotion elicited by observing a need situation), and experienced *incidental* guilt (a negative emotional state elicited by something else than the need situation) have been shown to increase subsequent helping (e.g., Cialdini et al., 1987; Dovidio et al., 1991; Sachdeva et al., 2009; Cryder et al., 2012; Rotella and Richeson, 2013).

Central for the current article, Baumeister et al. (2007) convincingly argue that the anticipation of emotions is more important than the experience of emotions as a driver of behavior (see also Mellers et al., 1999; Malti and Krettenauer, 2013). In the case of guilt, although experienced guilt after doing something immoral provides salient feedback about one's actions, it may be argued that one of the main functions of guilt is to “teach a lesson” and instill a strong affective cue that can guide future behavior. The concept of *anticipated guilt if not helping* therefore plays a central role in this study. Studies have shown that anticipated guilt positively predict many types of prosocial behavior such as donation intentions (Basil et al., 2008), recycle-intention (Elgaaied, 2012), as well as intention to donate organs and bone marrow (Lindsey, 2005; Wang, 2011). Also, anticipated guilt if not helping is higher if reading about one identified victim than if reading about many statistical victims (Lindsey, 2005; Lindsey et al., 2007) and putting an animated face on a tree or a bulb increase both anticipated guilt if not complying, and actual pro-environment behavior (Ahn et al., 2014).

### Helping as emotion regulation: avoiding negative emotions or approaching positive emotions?

For the person experiencing it, guilt undoubtedly has a negative valance, so helping in order to relieve one's currently experienced guilt, and helping in order to prevent feeling guilty in the future is exclusively about avoiding something bad. Importantly, despite its name, the well-known Negative state relief model is not only about relieving negative emotions (going from a negative to a neutral state) but also includes producing positive emotions (going from a neutral to a positive state; Cialdini and Kenrick, 1976; Baumann et al., 1981; Cialdini et al., 1987). The motivation to help is claimed to be about hedonism, improving ones mood or “feeling better,” but it is important to notice that one can achieve this either by avoiding the negative (i.e., relieving and avoiding negative emotions) or by approaching the positive (i.e., retaining and approaching positive emotions). The distinction between avoiding the negative and approaching the positive has been much discussed in general terms (e.g., Bandura, 1986; Carver and Scheier, 1990; Roseman et al., 1990). For example, it has been argued that emotional self-regulation can have either a promotion focus where the question is whether one will gain or not gain (e.g., if you do good, you will win money), a prevention focus where the question is if whether one will lose or not lose (e.g., if you do good you don't lose money), or both (Higgins, 1998). Although avoiding the negative and approaching the positive have been distinguished in other domains both when it comes to actual reactions (e.g., Higgins et al., 1997) and when it comes to anticipated reactions (e.g., Onwezen et al., 2014; Hur and Jang, 2015), they have, with a few exceptions, not been empirically separated in the prosocial decision making literature. The distinction between helping in order to avoid negative emotional consequences and helping in order to approach positive emotional consequences for oneself is central for this paper.

### Warm glow, anticipated warm glow, and helping

The positive equivalent to guilt is *warm glow* (Andreoni, 1990; Dunn et al., 2014). Warm glow is here conceptualized as a pleasurable (positive) self-directed emotional reaction that arises as a result of acting in a good or moral way. Warm glow in this definition includes aspects of positive empathy (i.e., being happy because someone else is happy; Andreychik and Migliaccio, 2015), as well as the self-conscious emotion of pride (i.e., being happy because of causing a personally valued outcome, e.g., Tangney and Tracy, 2012). In the field of prosocial behavior, it has been shown that people who are given a sum of money and told to spend the money on others (i.e., helping) report feeling happier than people who are told to spend it on themselves (Dunn et al., 2008, 2014). According to the empathic joy hypothesis, an important motivator of helping is the amount of joy (warm glow) the helper receives from helping (Smith et al., 1989). The amount of warm glow one receives from helping can be affected both by the amount of feedback (less warm glow if one does not learn how the act of helping benefitted the victim; Smith et al., 1989) and from the relative impact ones help could do for the victim (more warm glow if one can make a large proportional impact; see Duncan, 2004).

Warm glow can refer to a currently experienced positive emotion which can influence future behavior (incidental warm glow) but the focus here is on *anticipated warm glow if helping* (e.g., Västfjäll et al., 2015). When faced with a possibility to help, most people imagine both how they would feel in case they decided not to help, and in case they decided to help. Previous studies show that anticipated warm glow if helping is reduced in situations where the helper is made aware of victims she cannot personally help (Västfjäll et al., 2015), when the help is inefficient (Bekkers, 2010), when there is more than one victim (Västfjäll et al., 2014) and in situations where one is far from solving the problem (Cryder et al., 2013). In a study investigating which types of motivation that predict future blood donations it was found that anticipated warm glow, but not anticipated tangible rewards, altruistic motivations or anticipated health benefits for oneself and one's family, predicted future blood donation intentions (Ferguson et al., 2008).

Although both anticipated guilt if not helping and anticipated warm glow if helping have often been included as an underlying mechanism of helping in earlier research, only a few previous studies have, to our knowledge, tested anticipated negative emotions if not helping and anticipated positive emotions if helping side by side. For example, Cryder et al. (2013) and Västfjäll et al. (2015) only measured anticipated warm glow if helping, whereas, e.g., Lindsey (2005) and Ahn et al. (2014) only measured anticipated guilt if not helping. Dickert et al. (2011) did in fact measure both the anticipation of feeling better if helping and the anticipated regret if not helping but aggregated these into a single variable called mood management. Likewise, Ferguson et al. (2012) aggregated anticipated positive feelings associated with donating blood and anticipated negative emotions associated with not donating blood into the same motivation-class.

A notable exception is found in research done by Krettenauer and colleagues. In one study they found that negative emotion expectancies (≈ anticipated guilt) was stronger when imagining harming than if imagining not helping, but that positive emotion expectancies (≈ anticipated warm glow) was stronger if imagining helping than if imagining not harming (Krettenauer and Johnston, 2011). Another study found similar results for both self-oriented (e.g., pride and guilt) and other-oriented (e.g., admiration and anger) positive and negative emotions, and also cultural differences in that whereas Canadian adolescents found it much more obligatory to refrain from harming than to engage in helping, this distinction was not as strong for Chinese adolescents (Krettenauer and Jia, 2013).

The research question to be tested in this paper is whether or not anticipated guilt if not helping and anticipated warm glow if helping should be considered two separate constructs rather than part of a single “mood management” construct in helping motivation. The research question was arrived to after reviewing the literature on mood improvement and finding seemingly contradictory patterns of guilt and warm glow. For example, while anticipated guilt if not helping is higher in situations where the victim is important for the helpers well-being (e.g., more anticipated guilt if not helping a person that later will allocate money between the two of you; Baumeister et al., 1995; Nelissen, 2014), positive emotions such as perceived meaningfulness of helping is higher in situations where helping involves some personal discomfort (c.f. moral martyrdom; Olivola and Shafir, 2013), and becomes more important in helping decisions where people are asked to help with time rather than with money (c.f. the time-ask effect, Liu and Aaker, 2008).

If anticipated guilt and anticipated warm glow are tapping in to the same underlying “mood management-construct,” they will be similarly influenced by situational factors (e.g., situations that elicit strong anticipated guilt if not helping will also elicit strong anticipated warm glow if helping). On the other hand, if anticipated guilt and anticipated warm glow are measuring different types of reactions, they will at least sometimes be differently influenced by situational factors (e.g., situations that elicit relatively strong guilt if not helping will elicit relatively weak warm glow if helping and vice versa). In other words, we expect that a change in the helping situation can increase anticipated guilt if not helping but at the same time decrease anticipated warm glow if helping. The next question concerns what types of situational differences that potentially could influence anticipated guilt and anticipated warm glow in opposite directions?

## Having a responsibility to help

As noted above, the studies by Krettenauer and Johnston (2011) and Krettenauer and Jia (2013) clearly separated positive and negative reactions and found that they were differently influenced by proscriptive morality (i.e., do not harm) and prescriptive morality (i.e., help, Janoff-Bulman et al., 2009). However, whereas their studies focused primarily on the difference between acts and omissions, our study focuses only on prescriptive morality (participants imagine their reactions if helping and/or if not helping), but varies the degree of responsibility to help.

People help more when they believe they have a personal responsibility to do so than when they do not (Baumeister et al., 1994; Jeske, 2008; Rai and Fiske, 2011). Batson (2011) acknowledges that helping can be motivated by moral principles and that doing ones responsibility is a kind of moral principle. It has further been shown that although empathic feelings predict helping, its predictive power drops in magnitude and often loses significance when controlling for the perceived responsibility to help (Wilhelm and Bekkers, 2010).

Responsibility and guilt have previously been intimately connected (Basil et al., 2006; Berndsen and Manstead, 2007; Gebauer et al., 2008; Zimmermann et al., 2011). Hoffman (1982) argues that to feel guilt, both empathic distress and some degree of self-attribution of responsibility for the victim's suffering are necessary and Baumeister et al. (1994), suggest that although perceived responsibility is not a requirement for guilt, it is an important determinant of the magnitude of experienced guilt. Guilt mediated helping done by a transgressor (i.e., a person accepting a causal responsibility) but other factors mediated helping done by non-transgressors in a study by Regan (1971). In sum, not helping in a situation where one perceives a responsibility to help almost always leads to feelings of guilt whereas not helping in a situation where one does not perceive a responsibility hardly elicit any guilt at all.

The relation between responsibility and warm glow has not been as well researched. Still, Weinstein and Ryan (2010) found that people experience less well-being after helping if the helping decision is partially outside their own control. In one of their studies, donating more rather than less money was related to an improved mood if the helping was autonomous but to a slightly worsened mood if the helping was controlled. In other words, if people feel obligated to help (by others or by ones internalized moral standards), warm glow reactions will be weaker than if the choice to help is totally up to the helper (see also Dunn et al., 2014). Likewise, Harbaugh et al. (2007) showed that the reward-center in the brain is activated when voluntary giving is performed, but less so when obligatory giving is performed.

Taken together, these results suggest that guilt and warm glow could be influenced in different ways when manipulating situational aspects related to personal responsibility. In high-responsibility situations, people should anticipate feeling strong guilt if not helping but weak warm glow if helping. On the other hand, in low-responsibility situations people should anticipate feeling weak guilt if not helping but strong warm glow if helping.

## Overview of the studies

We used four studies to test our hypothesis. The studies were similar in the sense that all participants read several scenarios each describing a different hypothetical helping situation. For each scenario, they were told to imagine themselves in the role of the potential helper and asked to rate their anticipated emotions if helping (anticipated warm glow) and/or if not helping (anticipated guilt). Each helping scenario was written in at least one high-responsibility version and one low-responsibility version, and pilot-studies confirmed that the perceived responsibility to help varied as a function of scenario-version. The main difference between the four studies was that the two different factors (type of anticipated emotion and responsibility-version) were rated and evaluated by participants either separately or jointly (c.f. General evaluability theory Hsee and Zhang, 2010).

### Different responsibility-manipulations

Several situational aspects are known to influence the perceived responsibility to help, and in order to increase generalizability we included many types of responsibility-manipulations. In each included helping-scenario, we manipulated a specific situational aspect assumed to influence the degree of perceived responsibility. The situational aspects and scenario-specific hypotheses are briefly explained below.

#### Effort

Not only the perceived benefit for the victim but also the perceived personal cost of helping influences responsibility. People perceive a high responsibility to help if the helping behavior is effortless but perceive a low responsibility to help if the helping behavior is effortful. Effort of helping can be manipulated by varying the time or energy it would require to help, or by varying one's available resources. We predict that anticipated guilt if not helping will be higher when helping is relatively effortless (e.g., helping is quick, cheap and done with money rather than time), but that anticipated warm glow if helping will be higher when helping is relatively effortful (c.f. Olivola and Shafir, 2013).

#### Cause of problem

A potential helper who has caused the victim's need situation (either by negligence or by mistake) will perceive herself as more responsible to help compared to if the need situation was caused by bad luck or by the victim herself. We thus predict that anticipated guilt if not helping will be higher when the potential helper caused the problem, but that anticipated warm glow if helping will be higher when the problem was caused by bad luck or by the victim^1^.

#### Social closeness

People believe they have a greater responsibility to help victims that are socially close to them (e.g., relatives, friends, countrymen) than victims that are socially distant (Baumeister et al., 1994; Baron et al., 2013; Erlandsson et al., 2015). We thus predict that anticipated guilt if not helping will be higher when the person in need is socially close, but that anticipated warm glow if helping will be higher when the person in need is socially distant.

#### Promises, expectations and requests

Making a promise entails a responsibility to keep it (Vanberg, 2008), and one reason for this is that promises increase others' expectations. In fact, even unfounded expectations from others can increase the perceived responsibility to comply (Baumeister et al., 1995). Also, an explicit request is an obvious way of communicating an expectation, suggesting that requests increase the perceived responsibility to help. We thus predict that anticipated guilt if not helping will be higher when the victim expects to be helped (e.g., unfounded expectation, promise-based expectation or request-based expectation), but that anticipated warm glow if helping will be higher when the victim does not expect to be helped.

#### Bystanders

The traditional explanation for the famous bystander effect (Fischer et al., 2011) is diffusion of responsibility meaning that for each additional bystander, the responsibility to help is distributed among many thus reducing one's personal responsibility (Cryder and Loewenstein, 2012). We thus predict that anticipated guilt if not helping will be higher when there are no other potential helpers, but that anticipated warm glow if helping will be higher when there are many other potential helpers.

## Ethical considerations

Participation was anonymous and all participants were informed beforehand what kind of task they would be doing. They were also told that they could stop their participation at any time without explanation. This created an informed consent. The study was tacitly approved by an in-house ethics committee at the Department of Psychology at Lund University but because the informed consent, because the hypothetical and non-intrusive nature of the study and because participants were over 18 years old, no formal ethics committee were required to review the study.

## Study 1

Study 1 was conducted to test if anticipated guilt if not helping and anticipated warm glow if helping are similarly or differently influenced when manipulating responsibility in several different ways. Two separate studies were conducted (Study 1a and Study 1b). The methodology of the studies was identical but the included situations and type of responsibility-manipulations differed. Participants were told that their task was to read and imagine six hypothetical scenarios presented on separate pages, and to respond to the questions following each scenario. Each participant read three scenarios written in the high-responsibility version and three scenarios written in the low-responsibility version. Half of the participants were asked to rate their anticipated guilt if not helping in each scenario. The other half was asked to rate their anticipated warm glow if helping in each scenario. Thus, both type of anticipated emotion and responsibility-version was evaluated separately in Study 1.

## Study 1A

### Method

One-hundred eighty six Swedish students participated by filling out a paper and pen questionnaire. Fifteen participants were excluded for missing more than one question in the questionnaire or for talking to other people during participation^2^. The remaining participants (83 female, 78 male, and 10 unknown) had a mean age of 22.02 years (*SD* = 3.09).

The first manipulation concerned type of anticipated emotion and was measured between-groups. Half of the participants were asked to imagine how they would feel if they did not help in each situation and to respond to three questions measuring their anticipated guilt if not helping: 1. I would feel guilty if I did not help, 2. I would have a bad conscience if I did not help, and 3. I would feel regret if I did not help (all Crombach's α'*s* > 0.89). The other half were asked to imagine how they would feel if they actually helped in each situation and to respond to three questions measuring their anticipated warm glow if helping: 1. I would experience a warm pleasurable feeling if I helped, 2. I would feel satisfied if I helped, and 3. I would feel that I did something very nice if I helped (all Crombach's α'*s* > 0.73). These ratings were done on seven-point Likert-scales where *0* = not at all and *6* = very much.

The second manipulation concerned degree of responsibility to help. Six helping-scenarios were written in one high-responsibility version and one low-responsibility version and each participant read three scenarios written in a high-responsibility version and three scenarios written in a low-responsibility version, but never both versions of the same scenario. The order of the scenarios and the version-order combinations were balanced in twelve different ways.

A pilot-study done on a separate sample after the main study (*N* = 101), confirmed that the perceived personal responsibility to help was significantly higher in the high-responsibility version than in the low-responsibility version in four scenarios and directionally but non-significantly higher in the high-responsibility version for the bystanders-scenario. One of the included scenarios did not pass the manipulation check so this scenario is not included in the analyses^3^. See Table 1 for a summary of the included scenarios and their respective high-/low-responsibility versions, and see Appendix 1 for all scenarios in full-text.

**Table 1:** **Summary of the included scenarios in Studies 1a (first five scenarios) and 1b (last five scenarios)**.

**Scenario name**	**Context**	**High- responsibility version**	**Low-responsibility version**	**Type of helping**
Expectation	Your grandmother who loved volunteering recently died	*Expectation*: You know that your grandmother expected you to start volunteering.	*No expectation*: You know that your grandmother expected you to follow your own heart.	Signing up to volunteer at a soup kitchen
Effort	You read that there is currently a short supply of your blood type.	*Small effort*: To donate blood would not take a lot of effort from your part (30 min)	*Big effort*: To donate blood would take a lot of effort from your part (8 h)	Donating blood
Request	You meet a foreign man at the train-station who missed the last bus to his destination due to no mistake of his own	*Request*: The foreign man gets anxious and explicitly ask you if you can pay for a taxi	*No request*: The foreign man does not ask you for anything	Paying a taxi for the man
Social Closeness	You ride on a bus on the way to your work. While riding, you see a young woman sitting on a bench crying hard.	*Relative*: The young woman is your cousin	*Stranger*: The young woman is someone you recognize from your local supermarket but have never spoken to	Jumping of the bus to see if you can help the girl (implying that you will be late for work)
Bystanders	You are in a hurry and bike through a park. You see a boy fall from a jungle gym. The boy has a nosebleed and cry heavily	*No bystanders*: No one expect yourself are close to the incident	*Bystanders*: In the distance, there is a group of other adults who soon will notice that the boy is injured	Stopping the bike to help the boy (implying that you will not arrive in time)
Type of helping	You see a friendly but very poor older man drop and lose some bills in the wind when entering the supermarket to buy food. He gets very sad	*Helping with money*: You consider helping by giving him some bills.	*Helping with time*: You consider helping by inviting him for dinner	Give him bills/invite him for dinner
Resources	You just won some money on a lottery. Later the same day you are approached by a fund-raiser from Red Cross asking you for a one time donation of 300SEK.	*Big resources*: You won a lot of money on the lottery (50.000 SEK)	*Small resources*: You won a small sum of money on the lottery (500 SEK)	Making a one-time donation of 300SEK
Cause (Money)	A woman at your job forgot her purse at work and the next day it was stolen by someone who entered the building. The woman lost a lot of money and it is approaching Christmas	*Your fault*: The thief entered the building through a window that you forgot to close	*Victims fault*: The thief entered the building through a window that the woman forgot to close	Lending the woman 5000SEK and paying her insurance deposit
Promise	You talk to an acquaintance at a party. The acquaintance will move to another city some weeks later and worries about carrying heavy stuff in stairs.	*Promise made*: At the party, you promised the acquaintance that you will help her moving	*No promise made*: At the party, you said that you were sorry but that you could not help her moving	Accepting to help her moving when she creates a Facebook event about it several weeks later
Cause (Time)	You are on your way to a movie-premiere when you collide with another car with two seniors. No one is injured but their car has a damaged suspension. They need to go to the airport but cannot get hold of a taxi.	*Your fault*: The crash was clearly your fault	*Victims fault*: The crash was clearly their fault	Driving them to the airport in your car (hence missing the movie-premiere)

### Results

Each participant's anticipated emotion for the high-responsibility versions and for the low-responsibility versions were aggregated. We then conducted a 2 × 2 Mixed ANOVA where type of anticipated emotion (anticipated guilt if not helping/anticipated warm glow if helping) was a between-group factor and responsibility level (high-/low-responsibility version) was a within-subject factor. The interaction was significant, *F*_(1, 169)_ = 26.91, *p* < 0.001, η^2^ = 0.137, indicating that anticipated guilt if not helping and anticipated warm glow if helping are differently influenced by the responsibility manipulations^4^. Planned *t*-tests showed that whereas anticipated guilt was clearly higher in the high-responsibility versions (*M* = 3.57, *SD* = 1.03) than in the low-responsibility versions [*M* = 2.61, *SD* = 1.16; *t*_(84)_ = 5.93, *p* < 0.001, Cohen's *d* = 0.80], anticipated warm glow was similar in the high-responsibility versions (*M* = 3.96, *SD* = 0.92) and in the low-responsibility versions [*M* = 3.97, *SD* = 0.78; *t*_(85)_ = −0.03, *p* = 0.978]. This indicates that people anticipate feeling much more guilt if not helping in high-responsibility situations than in low-responsibility situations but that the anticipated warm glow if helping is not influenced by the responsibility manipulation.

**Figure 1:**
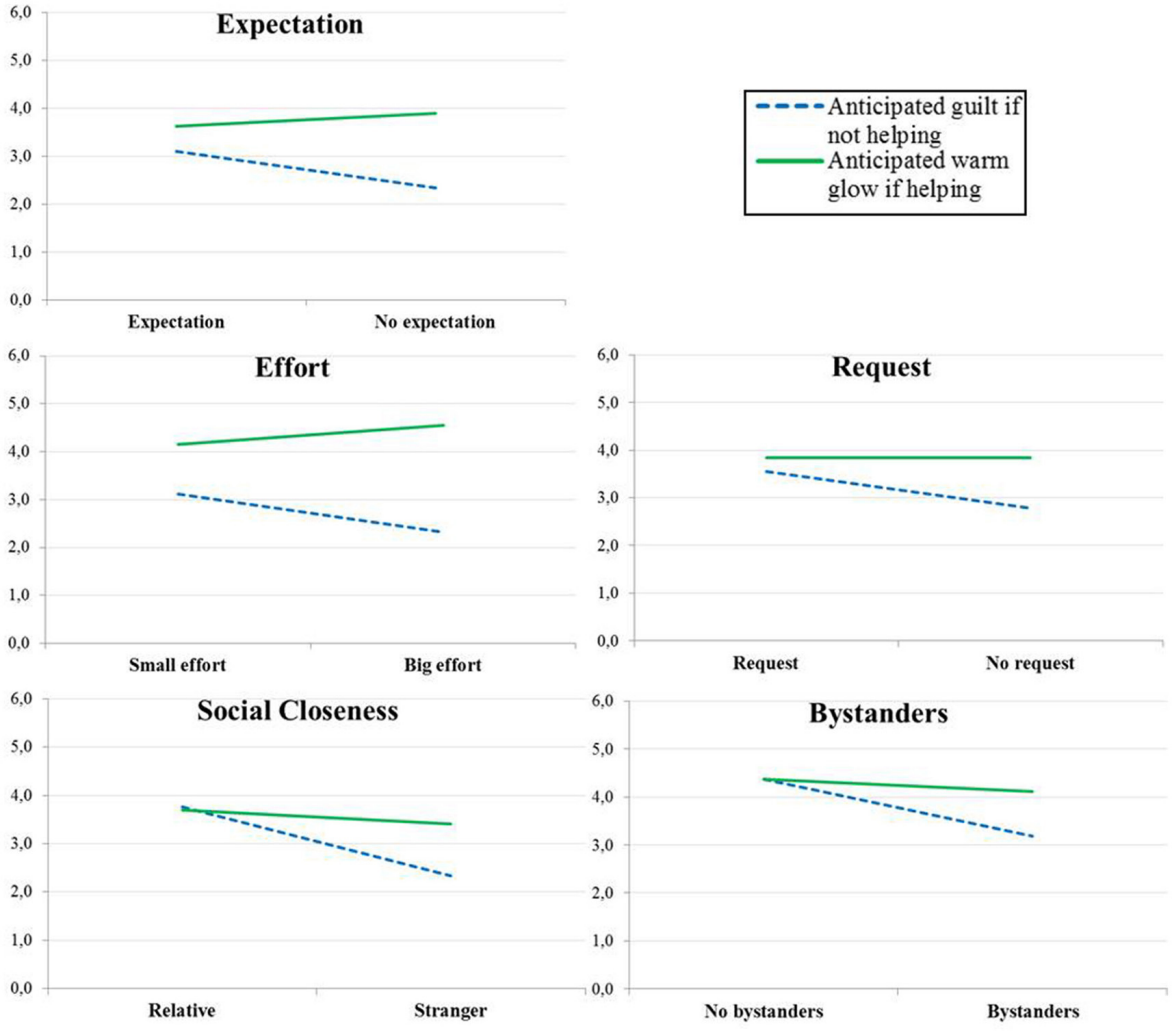
**Anticipated guilt if not helping and anticipated warm glow if helping in the five scenarios included in Study 1a**.

Looking at the five situations separately gives a similar picture (see Figure 1). The expected anticipated emotion type × responsibility version interaction was significant in four scenarios, and close to significance in one scenario^5^. Anticipated guilt was significantly higher in the high-responsibility version than in the low-responsibility version for all scenarios but anticipated warm glow never significantly differed when comparing the high- and low-responsibility versions (see Table 2).

**Table 2:** **Mean anticipated guilt and mean anticipated warm glow for each scenario in Study 1a (first five scenarios) and Study 1b (last five scenarios)**.

**Scenario name**	**Anticipated guilt if not helping**			**Anticipated warm glow if helping**		
	**High- responsibility version**	**Low- responsibility version**	***p***	**High- responsibility version**	**Low- responsibility version**	***p***
Expectation	3.10 (1.62)	2.33 (1.76)	0.041	3.63 (1.32)	3.90 (1.20)	0.328
Effort	3.11 (1.50)	2.32 (1.56)	0.019	4.16 (1.13)	4.55 (1.03)	0.096
Request	3.55 (1.39)	2.78 (1.55)	0.019	3.84 (1.32)	3.84 (1.14)	1.00
Social Closeness	3.77 (1.27)	2.34 (1.46)	0.000	3.69 (1.10)	3.41 (1.28)	0.282
Bystanders	4.37 (1.33)	3.19 (1.60)	0.000	4.37 (1.03)	4.11 (0.96)	0.230
Type of helping	3.81 (1.59)	2.74 (1.63)	0.002	4.41 (1.21)	4.24 (1.14)	0.533
Resources	3.34 (1.82)	2.49 (1.61)	0.022	3.61 (1.41)	3.70 (1.73)	0.799
Cause (Money)	4.05 (1.75)	2.99 (1.58)	0.004	3.94 (1.29)	4.28 (0.92)	0.179
Promise	3.70 (1.38)	1.57 (1.25)	0.000	3.67 (1.28)	3.46 (0.95)	0.408
Cause (Time)	4.05 (1.50)	2.80 (1.62)	0.000	4.04 (1.30)	3.82 (1.50)	0.481

## Study 1B

### Method

One-hundred eighty Swedish students participated but 10 were excluded for the same reasons as in Study 1a. The remaining 170 participants (76 female, 86 male, 8 unknown) had a mean age of 22.69 years (*SD* = 2.92). The procedure and conducted analyses was identical to Study 1a but the six scenarios and responsibility manipulations were different.

A pilot-study done on a separate sample after the main study (*N* = 108), confirmed that the perceived personal responsibility to help was significantly higher in the high-responsibility version than in the low-responsibility version in five scenarios^6^. See Table 1 for a summary of all the included scenarios and their respective high-/low-responsibility versions.

### Results

The interaction was significant, *F*_(1, 168)_ = 50.65, *p* < 0.001, η^2^ = 0.232, indicating that anticipated guilt if not helping (α's in the different scenarios ranging from 0.88 to 0.94) and anticipated warm glow if helping (α's ranging from 0.77 to 0.91) were differently influenced by the responsibility manipulations. Planned *t*-tests showed that anticipated guilt if not helping was clearly higher in the high-responsibility versions (*M* = 3.79, *SD* = 1.34) than in the low-responsibility versions [*M* = 2.50, *SD* = 1.30; *t*_(87)_ = 9.17, *p* < 0.001, *d* = 0.98], but that anticipated warm glow if helping was similar in the high-responsibility versions (*M* = 3.95, *SD* = 1.00) and in the low-responsibility versions [*M* = 3.88, *SD* = 1.05; *t*_(81)_ = 0.77, *p* = 0.444]. This again indicates that people anticipate feeling much more guilt if not helping in high-responsibility situations than in low-responsibility situations but that the anticipated warm glow if helping is not influenced by the responsibility manipulations.

**Figure 2:**
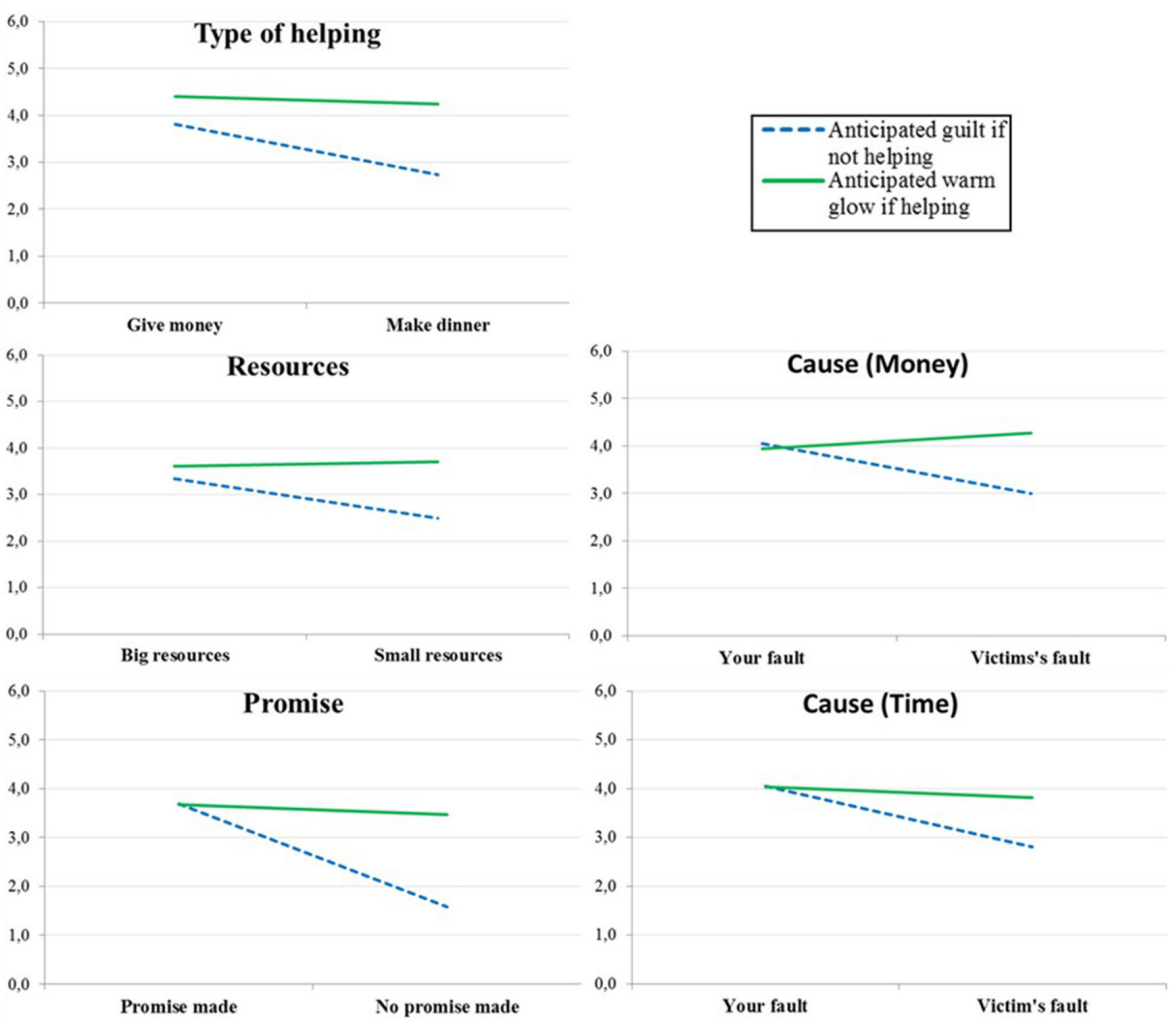
**Anticipated guilt if not helping and anticipated warm glow if helping in the five scenarios included in Study 1b**.

Looking at the five situations separately gives a similar picture (see Figure 2). The expected anticipated emotion-type × responsibility-version interaction was significant in four scenarios and close to significant in one scenario^7^. Anticipated guilt was significantly higher in the high-responsibility version than in the low-responsibility version for all scenarios but anticipated warm glow never significantly differed when comparing the high- and low-responsibility versions (see Table 2).

## Study 2

Whereas participants in Study 1 rated either only their anticipated guilt or only their anticipated warm glow, participants in Study 2 were told to imagine both helping and not helping and asked to rate both their anticipated guilt (if not helping) and their anticipated warm glow (if helping). In addition, whereas Study 1 included items measuring primarily the emotional aspects of anticipated guilt and anticipated warm glow, Study 2 measured guilt and warm glow both as anticipated self-directed emotions (i.e., how would you feel if helping/not helping) and in terms of anticipated self-image (i.e., what would you think about yourself if helping/not helping). Half of the participants in Study 2 thus rated their anticipated emotional guilt if not helping and anticipated emotional warm glow if helping whereas the other half rated their anticipated self-image guilt if not helping and their anticipated self-image warm glow if helping.

### Method

Swedish speaking participants were recruited on social media and asked to participate in an online survey. Two-hundred fifteen participants began filling out the online questionnaire and 194 completed it. Participants first read a brief description of anticipated guilt and anticipated warm glow and asked if they could understand these concepts. Two participants who did not understand the concept of anticipated guilt if not helping were excluded from the analysis. 116 female, 67 male, and 9 with unclassified sex were included (*M*_age_ = 31.69 years, *SD* = 9.93).

All participants read four helping scenarios used in Study 1 (Expectation, Effort, Cause[Money] and Request). Each participant read two scenarios in the high-responsibility version and the other two scenarios in the low-responsibility version. A scenario written in a high-responsibility version always followed a scenario written in the low-responsibility version and the other way around. Half of the participants read the scenarios in the order of (1) Expectation, (2) Effort, (3) Cause, and (4) Request, and the other half read the scenarios in the opposite order.

After each scenario, participants rated both anticipated guilt if not helping and anticipated warm glow if helping. Approximately half of the participants were asked two questions about the emotional type of anticipated guilt (“*I would feel guilty if I did not help*” and “*I would feel a bad conscience if I did not help*”) and the emotional type of anticipated warm glow (“*I would feel a warm sense of well-being if I helped*” and “*I would feel very satisfied if I helped*”). The other half were instead asked about the self-image type of anticipated guilt if not helping (“*I would see myself as a terrible person if I did not help*” and “*I would perceive myself to have done something immoral if I did not help*”) and the self-image type of anticipated warm glow if helping (“*I would perceive myself as an unusually good person if I helped*” and “*I would feel that I had done something very nice if I helped*”). All participants were first asked to imagine not helping and then asked to imagine helping. Ratings were done on seven-point Likert-scales where 1 = *not at all* and 7 = *very much*.

After reading the four scenarios and answering the four questions after each scenario, participants stated their sex, their current age and had the chance to write any comments, including if they had guessed the hypothesis.

### Results

We aggregated all four scenarios in order to test the hypothesis that anticipated guilt and anticipated warm glow are differently influenced by the responsibility manipulation. We conducted a 2 × 2 × 2 Mixed ANOVA. The first factor was type of reaction (anticipated guilt if not helping/anticipated warm glow if helping) measured within subjects. The second factor was scenario version (high/low responsibility) measured within subjects. The third factor was whether the anticipated reactions were emotional or related to self-image (measured between groups).

As expected, the type of reaction × responsibility-version interaction was significant, *F*_(1, 190)_ = 57.49, *p* < 0.001, η^2^ = 0.232, indicating that anticipated guilt and anticipated warm glow are differently influenced by the responsibility manipulation. The three-way interaction was not significant, *F*_(1, 190)_ = 2.26, *p* = 0.134, indicating that the type of reaction × responsibility version interaction does not change dramatically if one measures anticipated feelings (emotional) or anticipated thoughts about oneself (self-image). We therefore aggregated emotional and self-image guilt into a single anticipated guilt variable and emotional and self-image warm glow into a single anticipated warm glow variable, and used planned *t*-tests to examine if these variables were influenced differently by the responsibility manipulation. As expected, anticipated guilt was higher in the high-responsibility versions (*M* = 4.18, *SD* = 1.45) than in the low-responsibility versions [*M* = 3.44, *SD* = 1.44; *t*_(191)_ = 7.34, *p* < 0.001, *d* = 0.53]. In contrast, anticipated warm glow was lower in the high-responsibility versions (*M* = 4.94, *SD* = 1.31) than in the low-responsibility versions [*M* = 5.18, *SD* = 1.26; *t*_(191)_ = −3.01, *p* = 0.003, *d* = 0.22]. These results give further support to the idea that anticipated guilt if not helping and anticipated warm glow if helping are two different constructs, and shows that the two types of motivation can be affected in opposite directions when manipulating responsibility.

**Figure 3:**
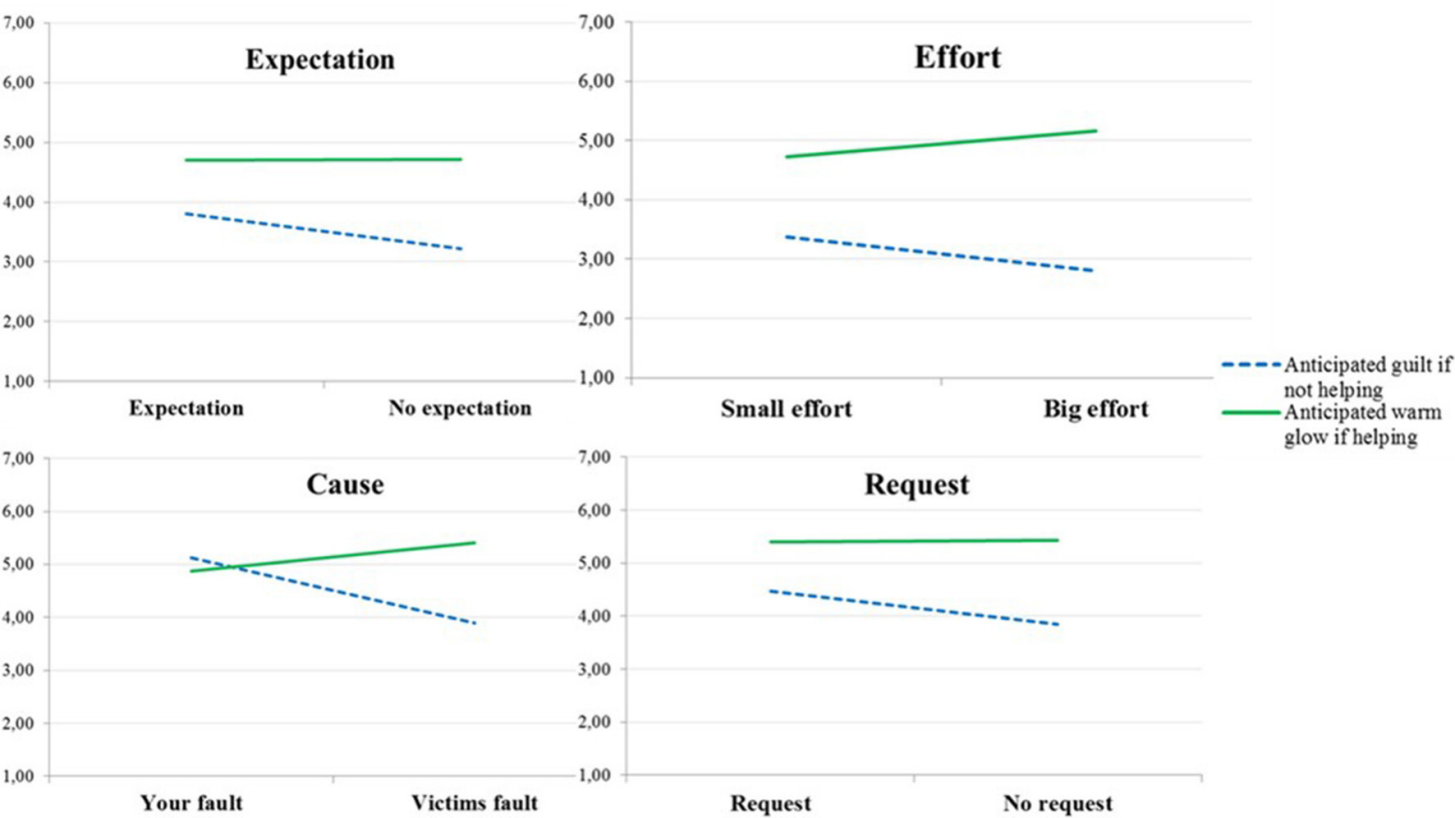
**Anticipated guilt if not helping and anticipated warm glow if helping in the four scenarios included in Study 2**.

We also investigated each scenario separately using the same 2 × 2 × 2 mixed ANOVA. The expected type of reaction × scenario version two-way interaction was significant for all scenarios^8^ (see Figure 3). The three-way interaction was not significant (all *F*'s < 1.3) for any of the scenarios and we therefore aggregated emotional and self-image guilt, and emotional and self-image warm glow. As can be seen in Table 3, whereas anticipated guilt was significantly *higher* in the high-responsibility than in the low-responsibility version in all four scenarios, anticipated warm glow was significantly *lower* in the high-responsibility version in the cause-scenario and marginally so in the effort-scenario.

**Table 3:** **Mean anticipated guilt if not helping and mean anticipated warm glow if helping for each scenario in Study 2**.

	**Anticipated Guilt if not helping**			**Anticipated Warm Glow if helping**		
	**High- responsibility version**	**Low- responsibility version**	***p***	**High- responsibility version**	**Low- responsibility version**	***p***
Expectation	3.80 (1.79)	3.22 (1.78)	0.024	4.71 (1.56)	4.71 (1.51)	0.980
Effort	3.37 (1.69)	2.81 (1.56)	0.018	4.73 (1.64)	5.16 (1.56)	0.068
Cause	5.13 (1.70)	3.89 (1.60)	0.000	4.49 (1.76)	4.87 (1.64)	0.013
Request	4.47 (1.51)	3.84 (1.68)	0.007	5.39 (1.29)	5.43 (1.42)	0.853

## Study 3

Whereas participants in the first studies read either a high-responsibility version or a low-responsibility version of each scenario, participants in Study 3 read five versions of each scenario. The five versions were presented so that responsibility decreased in each step. For each version, participants either rated their anticipated guilt if not helping or their anticipated warm glow if helping. As perceived responsibility to help became lower for each version, we expected anticipated guilt to decrease linearly but expected anticipated warm glow to increase linearly.

### Method

One-hundred eighteen prospective university students participated. 13 of these were removed from the analysis because they either did not complete the questionnaire, because they were under-aged or because they correctly guessed the hypothesis. The remaining 105 participants (67 women and 38 men) had a mean age of 22.11 years (*SD* = 8.62). Participants were recruited individually at a university fair and asked to complete a paper and pen questionnaire. They were told that the participation was anonymous and that they were going to be compensated with a lottery ticket worth 10 SEK for participating (≈ $1.2). All participants claimed to understand the concepts of anticipated guilt and anticipated warm glow.

All participants read four helping scenarios. Each scenario came with five different possible alternative endings (see Table 4 for summaries and Appendix 1 for full-text). Half of the participants were asked to imagine themselves not helping and to rate their anticipated guilt for each of the five alternative endings. The other half were asked to imagine actually helping and to rate their anticipated warm glow for each of the five alternative endings.

**Table 4:** **Summary of the included scenarios and alternative endings in Study 3 and 4**.

	**Scenarios**				
	**Effort (Both studies)**	**Victims fault (Study 3)**	**Fault (Study 4)**	**Bystanders (Both studies)**	**Closeness (Both studies)**
	You meet a foreign man at the train-station who missed the last bus to his destination. He asks you about how to go to an address which you know is rather far away	A classmate of yours has lost her bike and cannot afford buying a new one. You have a spare bike in your basement that you don't need and that you can lend	Your neighbor apartment has been broken in to and valuables have been stolen. Helping implies helping your neighbor clean up the mess in the apartment	You wear fancy clothes and walks quickly through a park in a hurry for a meeting. You see a woman fall off her bike and land in a puddle. She seems to have hurt her leg. Helping implies being late for the meeting and muddying your clothes	You ride on a bus on the way from school. While riding, you see a person having trouble carrying a heavy armchair. Helping implies jumping of the bus and carrying the armchair
**ALTERNATIVE ENDINGS**					
(a) high- responsibility	*Minimal Effort*: Explain that it is far and that he should take a cab, point toward the cab stand	*Totally innocent victim*: The bike was stolen despite having double locks and being kept inside	*Clearly your fault*: You let an unknown man into the staircase the night before.	*No bystanders*: You are alone in the park	*Extremely close*: The person carrying the armchair is your mother or father
(b)	*Small Effort*: Google the address on your cell phone and help him understand where it is and how to get there	*Slightly careless victim*: The bike was stolen when parked unlocked for a couple of minutes outside a store	*Maybe your fault*: You might have mistakenly forgot to check that the staircase door was locked properly	*One bystander*: You see that a jogger far away has also noticed the situation	*Very close*: The person carrying the armchair is your friend
(c)	*Some Effort*: Follow him to the cab stand, explain his situation for the cab driver and make sure the driver knows where to drive him	*Careless victim*: The bike was stolen when parked unlocked in the city for a couple of days	*Nobody's fault*: The neighbor was away travelling and you were inside your apartment the whole day before	*Some bystanders*: You see two young couples sitting on benches some distance away, and they has also noticed the situation	*Close*: The person is a classmate of yours
(d)	*Big Effort*: Follow him to the cab stand, explain his situation for the cab driver and pay the fare ($28)	*Somehow victim's fault*: The classmate simply forgot where the bike was parked when out partying	*Maybe the victim's fault*: The neighbor might have mistakenly forget to check that the staircase door was locked properly	*Several bystanders*: A group of softball-playing university students are close to the situation and have noticed the situation	*Somehow distant*: The person is a friend of a friend that you have met once before
(e) low- responsibility	*Extreme Effort*: Follow him to the cab stand, explain his situation for the cab driver, pay the fare ($28) and go with the cab in order to make sure he arrives safely.	*Clearly victims fault*: The classmate threw the bike from a balcony when partying	*Clearly victims fault*: The neighbor purposely left the staircase door open the night before	*Many eligible bystanders*: A group of police officers are close to the situation and have noticed the situation.	*Distant*: The person is someone you have seen in your local supermarket but do not know personally

*The order of the alternative endings was different in the two studies (high-responsibility ending first in Study 3, low-responsibility ending first in Study 4)*.

The four scenarios were presented to participants on separate pages in a balanced order. The layout for each scenario was that participants read a background-story and five alternative endings labeled *a* (the high-responsibility version) to *e* (the low-responsibility version)^9^. After each alternative ending, half of the participants were asked to rate their anticipated guilt if not helping in that specific circumstance whereas the other half were asked to rate their anticipated warm glow if helping in that specific circumstance. Responses were made on a Likert-scale ranging from *0* (none at all) to *10* = (extremely strong). After reading and responding to all five alternative endings of all four scenarios, participants read everything once again. This time around they were asked how probable it was that they would help for each alternative ending in all scenarios, with probability to help ranging from *0* (“*Not probable at all”*) to *10* (“Very probable”). This represented a crude measure of helping intention but as focus was on different types of anticipated emotions, the results relating to self-reported helping intentions are reported in Appendix 2.

### Results

For each scenario, we first tested if the two linear trends of anticipated guilt and of anticipated warm glow were different when varying responsibility over the five alternative endings. To do this, we conducted trend analyses with a mixed 5 × 2 ANOVA where the five alternative endings were measured within subjects and type of reaction (anticipated guilt if not helping/anticipated warm glow if helping) was measured between groups. It was expected that anticipated guilt and anticipated warm glow would be differently affected by the responsibility manipulation. We then investigated the linear trends of anticipated guilt and anticipated warm glow separately. It was expected that anticipated guilt would decrease linearly as the responsibility got lower in each alternative ending, but that anticipated warm glow would increase linearly as the responsibility got lower in each alternative ending. Because the obtained pattern differed substantially between the helping scenarios, each scenario is analyzed and reported separately in this study.

#### Effort scenario

**Figure 4:**
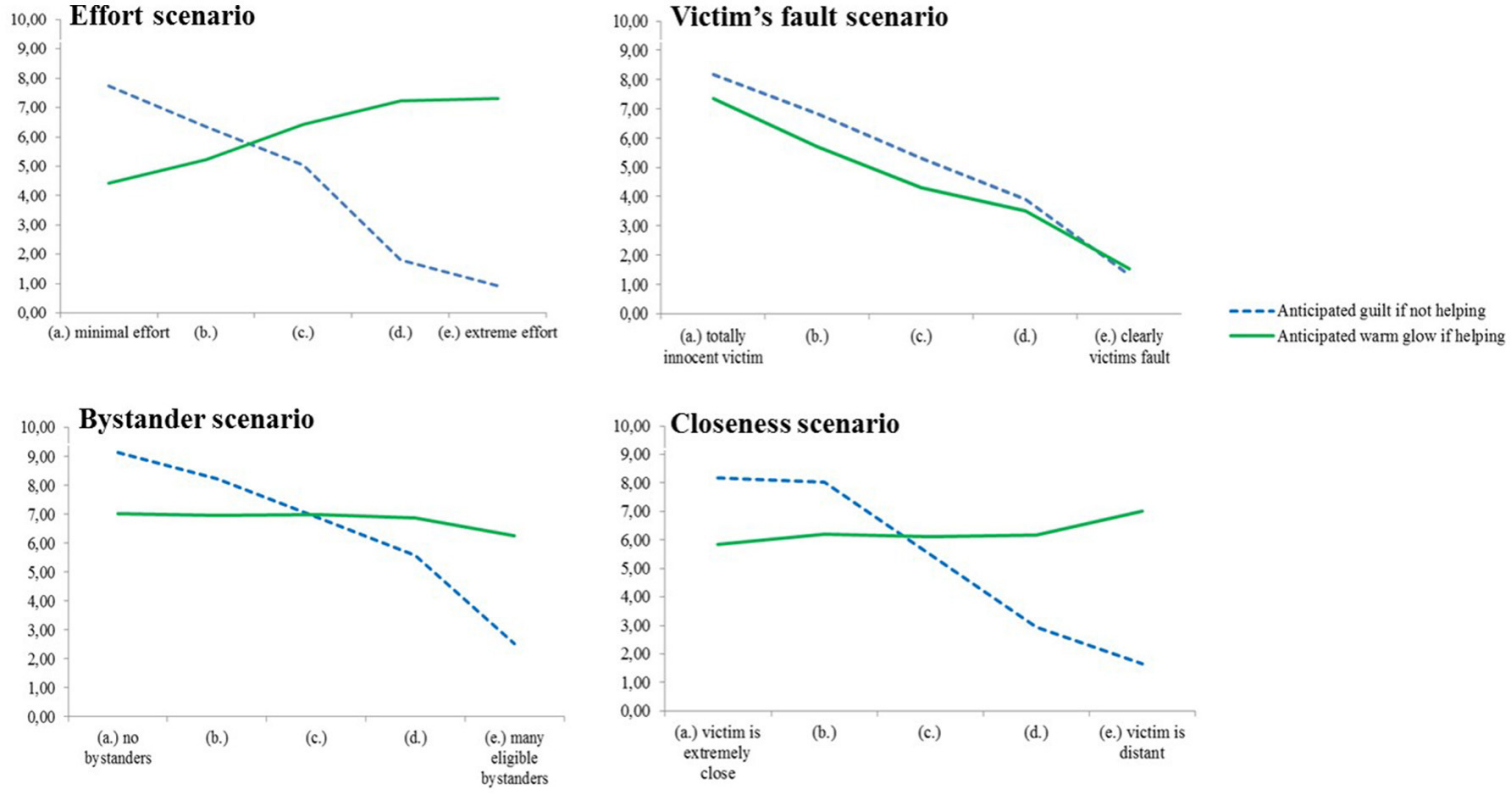
**Mean anticipated guilt if not helping and mean anticipated warm glow if helping in each of the alternative endings in the four scenarios in Study 3**. (a), Highest responsibility version; (e), Lowest responsibility version.

The interaction effect between the two linear trends of anticipated guilt and of anticipated warm glow whilst varying amount of effort was clearly significant: *F*_(1, 103)_ = 162.43, *p* < 0.001, η^2^ = 0.61, see Figure 4. The linear trend of anticipated guilt was clearly significant, *F*_(1, 49)_ = 377.78, *p* < 0.001, η^2^ = 0.89, and, as expected, people anticipated more guilt if not helping when the amount of effort to help was small than when the amount of effort to help was big. The linear trend of anticipated warm glow was also significant, *F*_(1, 54)_ = 19.78, *p* < 0.001, η^2^ = 0.27, but in the opposite direction. As expected, participants anticipated more warm glow if helping when the amount of effort to help was big than when the amount of effort to help was small.

#### Victim's fault scenario

No significant interaction effect between the two linear trends of anticipated guilt and of anticipated warm glow whilst varying the degree of victim's fault was found: *F*_(1, 103)_ = 3.47, *p* = 0.07, η^2^ = 0.03, see Figure 4. This suggests that anticipated guilt if not helping and anticipated warm glow if helping are similarly influenced by the victim's fault manipulation. The linear trend of anticipated guilt was clearly significant *F*_(1, 49)_ = 301.08, *p* < 0.001, η^2^ = 0.86, suggesting that people anticipate more guilt if not helping when the victim was totally innocent than when the victim caused her own problem. Against expectations, anticipated warm glow did not go in the opposite direction. In fact, people anticipated more warm glow if helping when the victim was totally innocent than when the victim caused her own problem, *F*_(1, 54)_ = 157.98, *p* < 0.001, η^2^ = 0.75. We try to explain this unexpected result in the discussion section.

#### Bystander scenario

The interaction effect between the two linear trends of anticipated guilt and of anticipated warm glow whilst varying number and eligibility of bystanders was clearly significant, *F*_(1, 103)_ = 109.64, *p* < 0.001, η^2^ = 0.52, see Figure 4. The linear trend of anticipated guilt was clearly significant, *F*_(1, 49)_ = 489.88, *p* < 0.001, η^2^ = 0.91, indicating that people anticipate more guilt if not helping when there are no bystanders than if not helping when there are many eligible bystanders. The linear trend of anticipated warm glow was not significant, *F*_(1, 54)_ = 2.03, *p* = 0.16, η^2^ = 0.04, indicating that people anticipate similar amounts of warm glow when helping alone as when helping in the presence of eligible bystanders.

#### Closeness scenario

The interaction effect between the two linear trends of anticipated guilt and of anticipated warm glow whilst varying social closeness was clearly significant, *F*_(1, 103)_ = 114.71, *p* < 0.001, η^2^ = 0.53, see Figure 4. The linear trend of anticipated guilt was clearly significant, *F*_(1, 49)_ = 208.75, *p* < 0.001, η^2^ = 0.81, indicating that people anticipate much more guilt if not helping when the victim is someone socially close than if the victim is someone socially distant. The linear trend of anticipated warm glow was in the opposite direction but not significant, *F*_(1, 54)_ = 2.58, *p* = 0.11, η^2^ = 0.05, indicating that people anticipate similar amounts of warm glow when helping in the absence and in the presence of eligible bystanders.

## Study 4

The aim of Study 4 was to replicate Study 3 but letting all participants rate both anticipated guilt if not helping and anticipated warm glow if helping for each alternative ending. In addition, we changed the Victim's fault scenario in Study 3 into a scenario where the target of fault for the situation went from the victim's fault via nobody's fault to the potential helpers fault.

### Method

The participants were 110 university students (81 women, 28 men, and 1 other) recruited around the university campus (*M*_age_ = 23.73 years, *SD* = 4.91). The procedure and materials were similar to those used in Study 3, but with the following changes: First and most importantly, anticipated guilt and anticipated warm glow were measured within subjects. Hence, for each alternative ending in each scenario, participants rated their anticipated guilt if not helping, their anticipated warm glow if helping and their self-rated probability to help on the same page. The order of presentation of the anticipated guilt and anticipated warm glow questions were balanced. Second, we changed the presentation-order of alternative endings to range from *a* (low responsibility) to *e* (high responsibility); i.e., the opposite order from Study 3. Third, the Victim's fault scenario in Study 3 was changed into a more general Fault scenario in Study 4 where the alternative endings ranged from *a* (problem was clearly accidentally caused by the victim) via *c* (nobody's caused the problem) to *e* (problem was clearly accidently caused by the potential helper; see Table 4 and Appendix 1).

### Results

The conducted analyses were identical to the analyses described in Study 3, with the exception that type of anticipated emotions was measured within subjects rather than between groups. Each helping scenario is again reported separately.

#### Effort scenario

**Figure 5:**
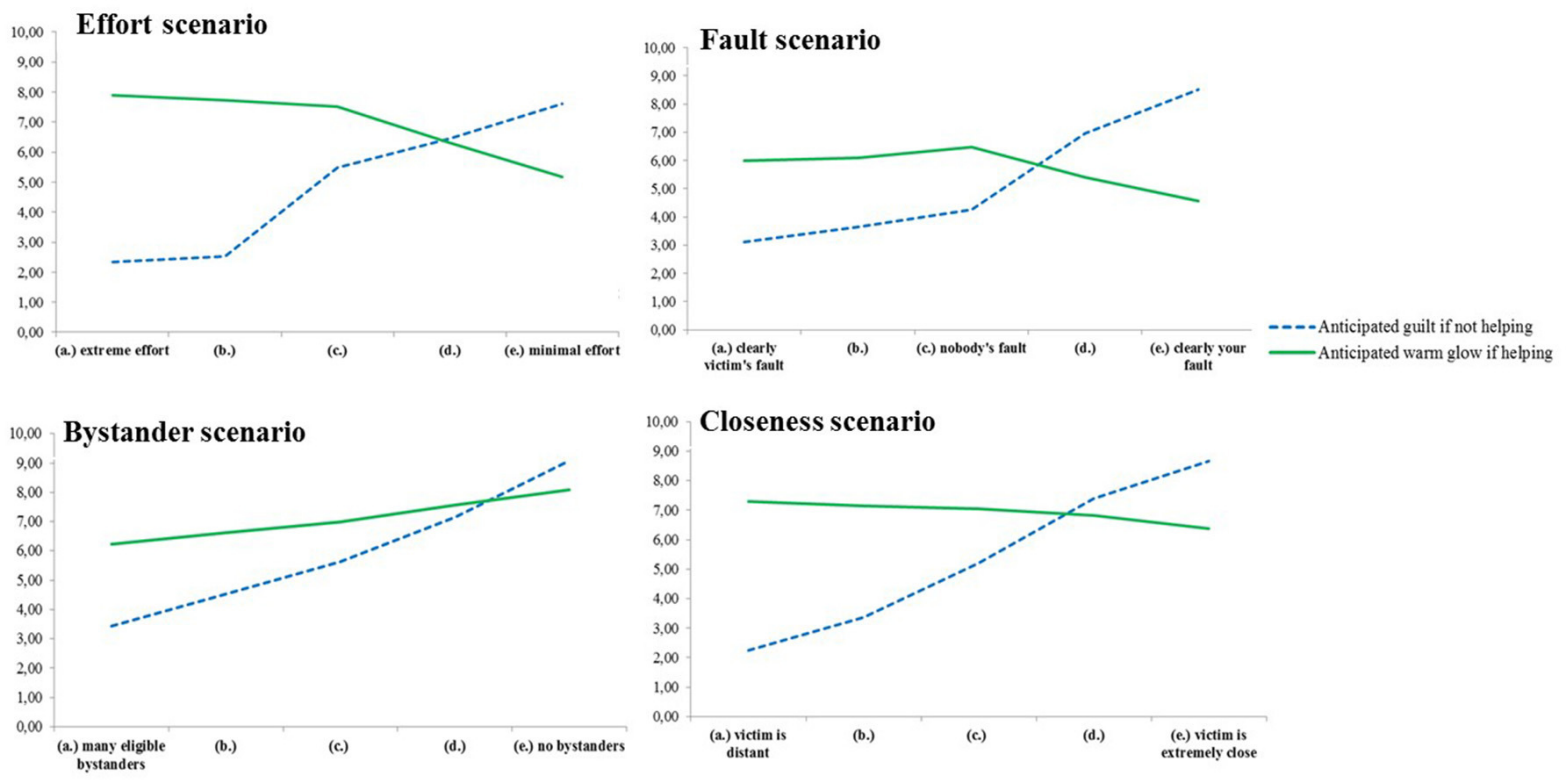
**Mean anticipated guilt if not helping and mean anticipated warm glow if helping in each of the alternative endings in the four scenarios in Study 4**. (a), Lowest responsibility version; (e), highest responsibility version.

The interaction effect between the two linear trends of anticipated guilt and of anticipated warm glow whilst varying the amount of effort was clearly significant, *F*_(1, 109)_ = 316.69, *p* < 0.001, η^2^ = 0.74, see Figure 5. The linear trend of anticipated guilt was significant *F*_(1, 109)_ = 368.66, *p* < 0.001, η^2^ = 0.77, showing that people anticipate more guilt if not helping when the effort to help is small than when the effort to help is big. The linear trend of anticipated warm glow was also significant but in the opposite direction, *F*_(1, 109)_ = 57.65, *p* < 0.001, η^2^ = 0.35, indicating that people anticipate more warm glow if helping when the effort to help is big than if helping when the effort to help is small.

#### Fault scenario

The interaction effect between the two linear trends of anticipated guilt and of anticipated warm glow whilst varying who caused the problem was clearly significant, *F*_(1, 105)_ = 216.06, *p* < 0.001, η^2^ = 0.67, see Figure 5. The linear trend of anticipated guilt was significant *F*_(1, 106)_ = 462.59, *p* < 0.001, η^2^ = 0.81, showing that people anticipate more guilt if not helping when they caused the problem than if the victim caused the problem. The linear trend of anticipated warm glow was also significant but in the opposite direction, *F*_(1, 105)_ = 14.11, *p* < 0.001, η^2^ = 0.12, indicating that people anticipate more warm glow if helping when nobody or the victim caused the problem than if helping when they caused the problem.

#### Bystander scenario

The interaction effect between the two linear trends of anticipated guilt and of anticipated warm glow whilst varying number and eligibility of bystanders was clearly significant, *F*_(1, 109)_ = 157.54, *p* < 0.001, η^2^ = 0.59, see Figure 5. This suggests that anticipated guilt if not helping and anticipated warm glow if helping are differently influenced by the bystander-manipulation. As expected, the linear trend of anticipated guilt was significant *F*_(1, 109)_ = 457.09, *p* < 0.001, η^2^ = 0.81, showing that people anticipate more guilt if not helping when there are no bystanders than if not helping when there are many eligible bystanders. Against expectations, the linear trend of anticipated warm glow was significant in the same direction, *F*_(1, 109)_ = 56.72, *p* < 0.001, η^2^ = 0.34, indicating that people anticipate more warm glow if helping when there are no bystanders than if helping when there are many bystanders. We try to explain this unexpected result in the general discussion.

#### Closeness scenario

The interaction effect between the two linear trends of anticipated guilt and of anticipated warm glow whilst varying social closeness of the victim was clearly significant, *F*_(1, 107)_ = 315.79, *p* < 0.001, η^2^ = 0.75, see Figure 5. The linear trend of anticipated guilt was significant *F*_(1, 108)_ = 727.95, *p* < 0.001, η^2^ = 0.87, showing that people anticipate more guilt if not helping when the victim is socially close than when the victim is socially distant. The linear trend of anticipated warm glow was also significant but in the opposite direction, *F*_(1, 107)_ = 6.40, *p* = 0.013, η^2^ = 0.06, indicating that people anticipate slightly more warm glow when helping someone socially distant than when helping someone socially close.

## General discussion

This study has demonstrated that anticipated guilt if not helping and anticipated warm glow if helping should be considered two different constructs rather than part of a single “mood maintenance” or “feeling better” construct when testing motivations of prosocial behavior. We did this by showing that anticipated guilt and anticipated warm glow were differently influenced when manipulating situational factors related to personal responsibility to help.

The four studies essentially tested the same thing but with different combinations of separate and joint evaluation of the two manipulations. Study 1 included 10 different responsibility-manipulations in order to give a broader understanding of how the anticipated emotions are influenced by various situational aspects. The expected interaction effect was significant or marginally significant for all 10 responsibility-manipulations. In Study 2 (where participants rated both types of reactions) guilt was significantly higher and warm glow significantly lower in the high-responsibility versions, hence suggesting that situational differences can influence anticipated guilt and anticipated warm glow in opposite directions. Study 3 and Study 4 manipulated personal responsibility in degrees within subjects rather than dichotomously between groups. Results differed depending of the type of responsibility manipulation used, but the expected pattern (i.e., compared to low-responsibility versions, high-responsibility versions elicit stronger anticipated guilt but weaker anticipated warm glow) emerged at least for the effort-manipulation, social closeness-manipulation and cause/fault-manipulation.

At the very least, these results imply that one cannot infer a person's anticipated warm glow if helping from knowing her anticipated guilt if not helping or the other way around. A specific helping situation can surely elicit both strong anticipated guilt if not helping and strong anticipated warm glow if helping, but our findings suggest that one can also imagine situations where anticipated guilt if not helping is relatively strong but anticipated warm glow if helping is relatively weak or vice versa.

### Discussion of the unexpected findings

We tested the hypotheses in five separate studies, and in each of them we included several types of responsibility-manipulations. For the most part, the results point in the same direction and support our idea that responsibility manipulations influence anticipated guilt and anticipated warm glow in different ways. Still, some unexpected findings emerged and these warrant further elaboration.

#### Separate or joint evaluations of anticipated guilt and anticipated warm glow

According to the General evaluability theory (Hsee and Zhang, 2010) our responses to a specific situation can vary a lot depending on if we evaluate only that situation (separate evaluation) or also another situation (joint evaluation). In this study, the hypothesis was tested using separate and joint evaluation of both responsibility-versions and of type of anticipated reaction. Both separate and joint evaluation comes with their respective inherent benefits and problems, so the fact that we found at least partial support for our hypotheses in all four studies strengthens the results of this study.

However, as we expected stronger anticipated warm glow in low-responsibility situations than in high-responsibility situations, it was unexpected to see that the responsibility-manipulation never influenced anticipated warm glow in Study 1 (and only for two of the scenarios in Study 3). One explanation of this finding is straightforward—that situational manipulations of responsibility influence anticipated guilt ratings but leave anticipated warm glow unaffected. Speculatively, this might not be limited to responsibility-manipulations specifically, but rather illustrate a general pattern that anticipated guilt is more context-dependent whereas anticipated warm glow is first and foremost dependent on individual differences.

Another explanation for the unaffected warm glow-ratings in Studies 1 and 3 is that some participants who were responding to the question about anticipated warm glow if helping, to some extent included anticipation of negative emotions if not helping in their responses (because it was not emphasized that they should focus exclusively on emotions if helping). If so, a positive effect of anticipated warm glow and a negative effect of anticipated guilt could nullify each other and create a flat line. In Studies 2 and 4, participants were asked both about their anticipated guilt if not helping and their anticipated warm glow if helping, thus making it much easier to distinguish the two types of anticipated reactions. This time, anticipated warm glow was indeed significantly affected by the responsibility-manipulation in the opposite direction. This indicates that the anticipated warm glow ratings in Studies 1 and 3, to some extent, could have been neutralized by participants also considering their anticipated guilt if not helping.

#### The cause manipulation in study 3

As predicted, anticipated guilt if not helping was always highest in situations where the potential helper had caused the need situation, and lowest in situations where the victim had caused the problem. When it comes to anticipated warm glow if helping, the result in Study 3 sticks out as confusing and at odds both with the hypothesis and with the results from the other studies in this article. Whereas, anticipated warm glow if helping was higher in the low-responsibility situation than in the high-responsibility situation in Study 1b and Study 2, the results from Study 3 showed a different pattern where anticipated warm glow was influenced in a similar way as anticipated guilt (highest when the problem was nobody's fault). We suggest that this pattern was due to two characteristics of the specific helping scenario used in Study 3. The victim in this scenario had lost her bike and the alternative endings ranged from the victim being totally innocent (having the bike stolen despite multiple precautions) to the victim causing the situation (throwing the bike from a balcony). This manipulation differed from the other included cause-manipulations first in that the manipulation ranged from nobody's fault to the victims fault thus not including any version where the potential helper caused the problem, and second, that the victim had caused her own problem by an intentional act (throwing a bike from a balcony) rather than by accident (e.g., forgetting to close a window or forgetting to close a door). We argue that although a small or non-existent responsibility to help in general imply more anticipated warm glow if helping, there are other factors that can influence the anticipated warm glow as well. For example, a sense of being exploited or a belief that one encourages negative behavior by helping is likely to cause anger and anger is most probably detrimental for warm glow. Although not measured in this study, it is reasonable to assume that people felt angry rather than sorry for the victim who caused her situation due to very reckless behavior but sorry rather than angry for the victim who caused her problem due to an honest mistake. Because we realized the inherent problems with the cause-manipulation in Study 3, we changed this scenario in Study 4 into a scenario similar to the one used in Studies 1b and 2, where the victim had caused the problem unintentionally and where the alternative endings ranged from clearly the victims fault to clearly the potential helpers fault with nobody's fault in the middle. The emerging pattern was in line with Studies 1b and 2 meaning that anticipated warm glow if helping was the lowest when the problem was caused by the potential helper. However, anticipated warm glow did not change if the problem was caused unintentionally by the victim or by chance, again suggesting that there might be other factors than only the level of perceived responsibility that influence anticipated warm glow.

#### The bystander manipulation

As predicted, the bystander manipulation clearly influenced anticipated guilt in all studies meaning that anticipated guilt if not helping was higher in situations where one is the only possible helper. Against our expectations (but in line with a prediction by Andreoni, 1995, p. 13), the bystander manipulation did not influence anticipated warm glow in the opposite direction. In Study 1a and 3, anticipated warm glow was not significantly affected by the presence of other helpers and in Study 4, anticipated warm glow was affected in the same direction as anticipated guilt (i.e., more anticipated warm glow if one is the only possible helper). One possible explanation comes from a study about “the embarrassed bystander” where it is suggested that embarrassment can act as an inhibitor to the decision to help in social situations (Zoccola et al., 2011). In this regard, it is again possible to assume that although a non-existent personal responsibility to help in general imply much anticipated warm glow if helping, other factors such as embarrassment can decrease anticipated warm glow (see Tangney and Tracy, 2012). If a potential helper doubts her own capabilities, helping a victim in front of others can be a more stressful and unpleasant event for her, as her actions will likely be evaluated by the bystanders.

## Theoretical implications

The primary aim of this study was to show that anticipation of positive emotions if helping is not the same as anticipation of negative emotions if not helping. It is possible to anticipate feeling very bad after not helping but simultaneously anticipate no positive feelings after helping or vice versa. Both anticipated guilt and anticipated warm glow have been included in previous studies and shown to motivate actual helping, but up until now the two types of motivation have rarely been properly distinguished and investigated side by side in the literature about helping decisions. Some researchers have in fact included both the approaching of warm glow and the avoidance of guilt as motivations for helping, but aggregated these into a single type of motivation (mood management in Dickert et al., 2011, or warm glow in Ferguson et al., 2012). Many other researchers have measured only one type of anticipated emotion (i.e., either anticipated guilt if not helping *or* anticipated warm glow if helping). As mentioned above, a potential risk with this procedure is that participants who are responding to questions about anticipated warm glow if helping might also consider anticipated guilt if not helping thus contaminating their warm glow ratings. For this reason, we suggest that future studies investigating prosocial motivations include both a measure of anticipated negative emotions if not helping and a measure of anticipated positive emotions if helping. The purpose of this study was not primarily to contribute to the emotion and emotion regulation literature (where this distinction usually is more clear), but rather to suggest that researchers on prosocial motivations include measures of both anticipated personal consequences if not helping and of anticipated personal consequences if helping.

In two studies that explicitly separated anticipated guilt and anticipated warm glow, it was found that Canadian adolescents expected more negative emotions (e.g., guilt) if acting antisocially than if not acting prosocially but that they expected more positive emotions (e.g., warm glow) if acting prosocially than if not acting antisocially (Krettenauer and Johnston, 2011; Krettenauer and Jia, 2013). As noted by the authors, the negative responsibility to refrain from harming is in general perceived as stronger than the positive responsibility to help. We agree, but argue that responsibility to help can vary a lot depending on the situation. For example, deciding whether or not to donate a large sum of money to a charity rally benefitting unknown victims (as in the scenario described in Krettenauer and Jia, 2013, p. 354) is a good example of a low-responsibility helping decision. The authors find that anticipated warm glow is more important than anticipated guilt for activating prosocial behavior using this and other scenarios (not printed in the article). We think their conclusion is true if the helping behavior is seen as non-obligatory (as in their example scenario) but suggest that the opposite might be true when helping is seen as obligatory. One could even argue that while a decision not to help in low-responsibility situations will be consistently perceived as an omission, a decision not to help in a high-responsibility situation might be perceived as an action despite still being an omission (for a related discussion see e.g., Knobe, 2010).

Another question concerns whether anticipated guilt and warm glow should be seen as purely egoistic reasons for helping or if there also are altruistic elements in these motivations? According to Batson (2011), helping behavior motivated by anticipated guilt and/or by anticipated warm glow is egoistic as the focus is on oneself. On the other hand, if the reason for deciding to help is empathic concern, and if anticipated guilt and/or anticipated warm glow are merely foreseen side-effects, then the motivation could be altruistic even if one's decision also implies less guilt or more warm glow for the helper. In practice however, it seems nearly impossible to know which emotion that is the reason for helping and which emotion that is a foreseen side-effect. Relatedly, it has been suggested that people who have self-related reasons for helping are perceived as less praiseworthy (e.g., Lin-Healy and Small, 2012; Newman and Cain, 2014), but at the same time, people experiencing warm glow after helping are perceived as more moral than people not experiencing it (Barasch et al., 2014). Possibly, people like helpers who do not anticipate, but who still experience positive emotions after helping, as this would indicate that they are altruistically motivated but still have the ability to experience typically human emotions. Importantly, these studies focused on positive personal consequences if helping. How people perceive guilt-motivated helping compared to warm-glow motivated helping is an interesting topic for future studies on social signaling.

## Limitations and suggestions for future studies

One limitation of the current study is that it has not focused on how anticipated guilt and anticipated warm glow influence actual helping. Anticipated negative emotions if not helping and anticipated positive emotions if helping have both been shown to predict helping intentions, and both dispositional guilt-motivation and dispositional warm glow-motivation significantly predict helping after controlling for the other (Gebauer et al., 2008). Helping intentions was in fact measured exploratory with a single item in Study 3 and 4 and the results are reported in Appendix 2. A quick look at the findings reveal that anticipated guilt is a clearly better predictor of helping intentions than anticipated warm glow in all helping scenarios, but there are no interpretable interactions with the different versions meaning that anticipated guilt is the relatively better predictor of helping intentions both in the low-responsibility situations and in the high-responsibility situations. Future studies should systematically investigate if there are some situations where actual helping behavior is primarily motivated by anticipated guilt and other situations where helping is primarily motivated by anticipated warm glow.

A different type of limitation is the broad definitions of guilt and warm glow. Admittedly, warm glow includes both the positive emotion elicited from others happiness (positive empathy; Andreychik and Migliaccio, 2015) and the positive emotion elicited from one's own desirable achievements (pride), and these positive emotions might not always predict reactions and behavior in the same way. Likewise, it can be argued that guilt is primarily internalized blame and that warm glow is primarily internalized praise. We agree but think these are very difficult to separate empirically. Also, our results from Study 2 suggested that the general pattern of results did not differ significantly if we measured guilt and warm glow as anticipated emotions or anticipated perceptions of one's self-image. Studies by Janoff-Bulman et al. (2009) show that people blame proscriptive violations (causing harm) more than prescriptive violations (failing to help), but that they praise proscriptive good deeds (not causing harm) less than prescriptive good deeds (helping), and that this pattern arise because proscriptive morality is our responsibility whereas prescriptive morality is not. These results correspond nicely to ours (and to Krettenauer and Johnston's 2011), but differ in the sense that we manipulated responsibility only within the prescriptive morality and that we focused on anticipated internalized blame (i.e., guilt) and anticipated internalized praise (warm glow) rather than actual blame and praise.

## Conclusion

It has long been acknowledged that one important motivation for helping others is to improve one's mood or to “feel better.” However, it has not been clear if this mood improvement primarily is about avoiding negative emotions, approaching positive emotions, or both. Therefore, this study tested if anticipated guilt if not helping and anticipated warm glow if helping were similarly or differently affected by different types of responsibility-manipulations. The results showed that anticipated guilt if not helping is higher in situations where the responsibility to help is high but that anticipated warm glow if helping often show the opposite pattern and is higher in situations where the responsibility to help is low. In sum, the results suggest that we anticipate guilt if not doing our duty but that we anticipate warm glow primarily when doing over and beyond our duty.

## Author contributions

AE, initiated the research project, designed the experiments, collected, and analyzed the data and wrote the manuscript; AJ, designed the experiments, collected and analyzed the data and wrote a bachelor thesis based on data from Studies 3 and 4 under supervision from AE; DV, wrote the manuscript.

### Conflict of interest statement

The authors declare that the research was conducted in the absence of any commercial or financial relationships that could be construed as a potential conflict of interest.

## Footnotes

^1^As noted by one of the reviewers, manipulating the cause of the problem is likely to influence not only anticipated guilt but also experienced guilt (i.e., a person who accidently caused harm to someone else could experience guilt because the harm already made, and also anticipate additional guilt if not helping. As experienced guilt was not controlled for in these studies, we cannot say to what extent it influenced the results. Results if removing the cause of problem-scenarios can be found in Appendix 2.

^2^After receiving the completed questionnaires in Study 1, the experimenter (AJ) discretely marked those where she believed the participant had openly discussed the questionnaire with others or where the participant seemed to fill in the questions in a haphazard manner. Importantly, the experimenter was unaware of any of the responses of the excluded participants when doing this. Exclusion was equally common in the anticipated guilt and anticipated warm glow conditions. Including the excluded participants did increase noise, but the results remained largely unchanged in all studies.

^3^In one scenario (existence of a social norm), the perceived responsibility to help was directionally smaller in the high-responsibility version (i.e., more than 90% of your co-workers donated one days salary to charity) than in the low-responsibility version (i.e., less than 5% of your co-workers donated one days salary to charity). This scenario was therefore not included in the main study analysis. A more detailed description of the pilot studies can be found in Appendix 2.

^4^In order to avoid making a long article even longer, only the interaction effects are reported in the main text. Inferential statistics of the accompanying main effects can be found in Appendix 2.

^5^Expectation, *F*_(1, 167)_ = 5.11, *p* = 0.025, η^2^ = 0.030; Effort, *F*_(1, 167)_ = 8.59, *p* = 0.004, η^2^ = 0.049; Request, *F*_(1, 167)_ = 3.40, *p* = 0.067, η^2^ = 0.020; Closeness, *F*_(1, 167)_ = 8.54, *p* = 0.004, η^2^ = 0.049; Bystander, *F*_(1, 167)_ = 5.74, *p* = 0.018, η^2^ = 0.033.

^6^In one scenario describing two boys going door to door to sell magazines, the perceived responsibility to help was directionally smaller in the presumed high-responsibility version (i.e., the boys have hardly sold anything yet) than in the presumed low responsibility version (the boys only need to sell two more until they have reached their goal). This scenario was therefore not included in the main study analysis. A detailed description of the pilot-study can be found in Appendix 2.

^7^Type of helping, *F*_(1, 166)_ = 4.35, *p* = 0.039, η^2^ = 0.026; Resources, *F*_(1, 166)_ = 3.47, *p* = 0.064, η^2^ = 0.020; Cause (Money), *F*_(1, 166)_ = 10.10, *p* = 0.002, η^2^ = 0.057; Promise, *F*_(1, 166)_ = 25.77, *p* < 0.001, η^2^ = 0.134; Cause (Time), *F*_(1, 166)_ = 5.12, *p* = 0.025, η^2^ = 0.030.

^8^Expectation, *F*_(1, 187)_ = 7.29, *p* = 0.008, η^2^ = 0.038; Effort, *F*_(1, 187)_ = 11.28, *p* = 0.001, η^2^ = 0.057; Cause, *F*_(1, 186)_ = 40.04, *p* < 0.001, η^2^ = 0.177; Request, *F*_(1, 186)_ = 3.96, *p* = 0.048, η^2^ = 0.021.

^9^Two small pilot studies were conducted prior to the main analysis in order to obtain five versions that together created strong linear effects on perceived responsibility for each scenario.

## References

[B1] Ahn H. K. Kim H. J. Aggarwal P. (2014). Helping fellow beings: anthropomorphized social causes and the role of anticipatory guilt. Psychol. Sci. 25, 224–229. 10.1177/0956797613496823 24192326

[B2] Andreoni J. (1990). Impure altruism and donations to public goods: a theory of warm-glow giving. Econ. J. 100, 464–477. 10.2307/2234133

[B3] Andreoni J. (1995). Warm-glow versus cold-prickle: the effects of positive and negative framing on cooperation in experiments. Q. J. Econ. 110, 1–21. 10.2307/2118508

[B4] Andreychik M. R. Migliaccio N. (2015). Empathizing with others' pain versus empathizing with others' joy: examining the separability of positive and negative empathy and their relation to different types of social behaviors and social emotions. Basic Appl. Soc. Psych. 37, 274–291. 10.1080/01973533.2015.1071256

[B5] Bandura A. (1986). Social Foundations of Thought and Action: A Social Cognitive Theory. Englewood Cliffs, NJ: Prentice-Hall Inc.

[B6] Barasch A. Levine E. E. Berman J. Z. Small D. A. (2014). Selfish or selfless? On the signal value of emotion in altruistic behavior. J. Pers. Soc. Psychol. 107, 393–413. 10.1037/a0037207 25133723

[B7] Baron J. Ritov I. Greene J. D. (2013). The duty to support nationalistic policies. J. Behav. Decis. Mak. 26, 128–138. 10.1002/bdm.768

[B8] Basil D. Z. Ridgway N. M. Basil M. D. (2006). Guilt appeals: the mediating effect of responsibility. Psychol. Mark. 23, 1035–1054. 10.1002/mar.20145

[B9] Basil D. Z. Ridgway N. M. Basil M. D. (2008). Guilt and giving: a process model of empathy and efficacy. Psychol. Mark. 25, 1–23. 10.1002/mar.20200

[B10] Batson C. D. (2011). Altruism in Humans. New York, NY: Oxford University Press.

[B11] Baumann D. J. Cialdini R. B. Kendrick D. T. (1981). Altruism as hedonism: helping and self-gratification as equivalent responses. J. Pers. Soc. Psychol. 40, 1039–1046. 10.1037/0022-3514.40.6.1039

[B12] Baumeister R. F. Stillwell A. M. Heatherton T. F. (1994). Guilt: an interpersonal approach. Psychol. Bull. 115, 243–267. 10.1037/0033-2909.115.2.243 8165271

[B13] Baumeister R. F. Stillwell A. M. Heatherton T. F. (1995). Personal narratives about guilt: role in action control and interpersonal relationships. Basic Appl. Soc. Psychol. 17, 173–198. 10.1080/01973533.1995.9646138

[B14] Baumeister R. F. Vohs K. D. DeWall C. N. Zhang L. (2007). How emotion shapes behavior: feedback, anticipation, and reflection, rather than direct causation. Pers. Soc. Psychol. Rev. 11, 167–203. 10.1177/1088868307301033 18453461

[B15] Bekkers R. (2010). Who gives what and when? A scenario study of intentions to give time and money. Soc. Sci. Res. 39, 369–381. 10.1016/j.ssresearch.2009.08.008

[B16] Berndsen M. Manstead A. S. R. (2007). On the relationship between responsibility and guilt: antecedent appraisal or elaborated appraisal? Eur. J. Soc. Psychol. 37, 774–792. 10.1002/ejsp.39727661548

[B17] Carver C. S. Scheier M. F. (1990). Origins and functions of positive and negative affect: a control-process view. Psychol. Rev. 97, 19–35. 10.1037/0033-295X.97.1.19

[B18] Cialdini R. B. Kenrick D. T. (1976). Altruism as hedonism: a social development perspective on the relationship of negative mood state and helping. J. Pers. Soc. Psychol. 34, 907–914. 10.1037/0022-3514.34.5.907 993985

[B19] Cialdini R. B. Schaller M. Houlihan D. Arps K. Fultz J. Beaman A. L. (1987). Empathy-based helping: is it selflessly or selfishly motivated. J. Pers. Soc. Psychol. 52, 749–758. 10.1037/0022-3514.52.4.749 3572736

[B20] Cryder C. E. Loewenstein G. (2012). Responsibility: the tie that binds. J. Exp. Soc. Psychol. 48, 441–445. 10.1016/j.jesp.2011.09.009

[B21] Cryder C. E. Loewenstein G. Seltman H. (2013). Goal gradient in helping behavior. J. Exp. Soc. Psychol. 49, 1078–1083. 10.1016/j.jesp.2013.07.003

[B22] Cryder C. E. Springer S. Morewedge C. K. (2012). Guilty feelings, targeted actions. Pers. Soc. Psychol. Bull. 38, 607–618. 10.1177/0146167211435796 22337764 PMC4886498

[B23] Dickert S. Sagara N. Slovic P. (2011). Affective motivations to help others: a two-stage model of donation decisions. J. Behav. Decis. Mak. 24, 361–376. 10.1002/bdm.697

[B24] Dovidio J. F. Piliavin J. A. Gaertner S. L. Schroeder D. A. Clark R. D. III. (1991). The arousal: cost-reward model and the process of intervention: a review of the evidence, in Prosocial Behavior, ed Clark M. S. (Newbury Park, CA: Sage), 86–118.

[B25] Duncan B. (2004). A theory of impact philanthropy. J. Public Econ. 88, 2159–2180. 10.1016/S0047-2727(03)00037-9

[B26] Dunn E. W. Aknin L. B. Norton M. I. (2008). Spending money on others promotes happiness. Science 319, 1687–1688. 10.1126/science.1150952 18356530

[B27] Dunn E. W. Aknin L. B. Norton M. I. (2014). Prosocial spending and happiness: using money to benefit others pays off. Curr. Dir. Psychol. Sci. 23, 41–47. 10.1177/0963721413512503

[B28] Elgaaied L. (2012). Exploring the role of anticipated guilt on pro-environmental behavior – a suggested typology of residents in France based on their recycling patterns. J. Consum. Mark. 29, 369–377. 10.1108/07363761211247488

[B29] Erlandsson A. Björklund F. Bäckström M. (2015). Emotional reactions, perceived impact and perceived responsibility mediate the identifiable victim effect, proportion dominance effect and in-group effect respectively. Organ. Behav. Hum. Decis. Process. 127, 1–14. 10.1016/j.obhdp.2014.11.003

[B30] Ferguson E. Atsma F. de Kort W. Veldhuizen I. (2012). Exploring the pattern of blood donor beliefs in first-time, novice, and experienced donors: differentiating reluctant altruism, pure altruism, impure altruism, and warm glow. Transfusion 52, 343–355. 10.1111/j.1537-2995.2011.03279.x 21848847

[B31] Ferguson E. Farrell K. Lawrence C. (2008). Blood donation is an act of benevolence rather than altruism. Health Psychol. 27, 327–336. 10.1037/0278-6133.27.3.327 18624597

[B32] Fischer P. Krueger J. I. Greitemeyer T. Vogrincic C. Kastenmüller A. Frey D.. (2011). The bystander-effect: a meta-analytic review on bystander intervention in dangerous and non-dangerous emergencies. Psychol. Bull. 137, 517–537. 10.1037/a0023304 21534650

[B33] Gebauer J. E. Riketta M. Broemer P. Maio G. R. (2008). Pleasure and pressure based prosocial motivation: divergent relations to subjective well-being. J. Res. Pers. 42, 399–420. 10.1016/j.jrp.2007.07.002

[B34] Haidt J. (2003). The moral emotions, in Handbook of Affective Sciences, eds Davidson R. J. Scherer K. R. Goldsmith H. H. (New York, NY: Oxford University Press), 852–870.

[B35] Harbaugh W. T. Mayr U. Burghart D. R. (2007). Neural responses to taxation and voluntary giving reveal motives for charitable donations. Science 316, 1622–1625. 10.1126/science.1140738 17569866

[B36] Hibbert S. Smith A. Davies A. Ireland F. (2007). Guilt appeals: persuasion knowledge and charitable giving. Psychol. Mark. 24, 723–742. 10.1002/mar.20181

[B37] Higgins E. T. (1998). Promotion and prevention: regulatory focus as a motivational principle. Adv. Exp. Soc. Psychol. 30, 1–46. 10.1016/S0065-2601(08)60381-0

[B38] Higgins E. T. Shah J. Friedman R. (1997). Emotional responses to goal attainment: strength of regulatory focus as moderator. J. Pers. Soc. Psychol. 72, 515–525. 10.1037/0022-3514.72.3.515 9120782

[B39] Hoffman M. L. (1982). Development of prosocial motivation: empathy and guilt, in Development of Prosocial Behavior, ed Eisenberg-Borg N. (New York, NY: Academic Press), 281–313.

[B40] Hsee C. K. Zhang J. (2010). General evaluability theory. Perspect. Psychol. Sci. 5, 343–355. 10.1177/1745691610374586 26162182

[B41] Hur J. Jang S. (2015). Anticipated guilt and pleasure in a healthy food consumption context. Int. J. Hosp. Manage. 48, 113–123. 10.1016/j.ijhm.2015.04.015

[B42] Janoff-Bulman R. Sheikh S. Hepp S. (2009). Proscriptive versus prescriptive morality: two faces of moral regulation. J. Pers. Soc. Psychol. 96, 521–537. 10.1037/a0013779 19254101

[B43] Jeske D. (2008). Special obligations, in The Stanford Encyclopedia of Philosophy (Fall 2008 ed.). ed Zalta E. N. Available online at: http://plato.stanford.edu/archives/fall2008/entries/special-obligations/

[B44] Knobe J. (2010). Person as scientist, person as moralist. Behav. Brain Sci. 33, 315–329. 10.1017/S0140525X10000907 20964912

[B45] Krettenauer T. Jia F. (2013). Investigating the actor effect in moral emotion expectancies across cultures: a comparison of Chinese and Canadian adolescents. Br. J. Dev. Psychol. 31, 349–362. 10.1111/bjdp.12012 23901847

[B46] Krettenauer T. Johnston M. (2011). Positively versus negatively charged moral emotion expectancies in adolescence: the role of situational context and the developing moral self. Br. J. Dev. Psychol. 29, 475–488. 10.1348/026151010X508083 21848742

[B47] Lindsey L. L. M. (2005). Anticipated guilt as behavioral motivation. Hum. Commun. Res. 31, 453–481. 10.1093/hcr/31.4.453

[B48] Lindsey L. L. M. Yun K. A. Hill J. B. (2007). Anticipated guilt as motivation to help unknown others: an examination of empathy as a moderator. Commun. Res. 34, 468–480. 10.1177/0093650207302789

[B49] Lin-Healy F. Small D. A. (2012). Cheapened altruism: discounting personally affected prosocial actors. Organ. Behav. Hum. Decis. Process. 117, 269–274. 10.1016/j.obhdp.2011.11.006

[B50] Liu W. Aaker J. (2008). The happiness of giving: the time-ask effect. J. Consum. Res. 35, 543–557. 10.1086/588699

[B51] Malti T. Krettenauer T. (2013). The relation of moral emotion Attributions to prosocial and antisocial behavior: a meta-analysis. Child Dev. 84, 397–412. 10.1111/j.1467-8624.2012.01851.x 23005580

[B52] Mellers B. Schwartz A. Ritov I. (1999). Emotion-based choice. J. Exp. Psychol. Gen. 128, 332–345. 10.1037/0096-3445.128.3.332

[B53] Miller C. (2010). Guilt and Helping. Int. J. Ethics 6, 231–252. 10.1007/s10892-010-9079-6

[B54] Nelissen R. M. A. (2014). Relational utility as a moderator of guilt in social interactions. J. Pers. Soc. Psychol. 106, 257–271. 10.1037/a0034711 24274086

[B55] Newman G. E. Cain D. M. (2014). Tainted altruism: when doing some good is evaluated as worse than doing no good at all. Psychol. Sci. 25, 648–655. 10.1177/0956797613504785 24403396

[B56] O'Keefe D. J. Figgé M. (1999). Guilt and expected guilt in the door-in-the-face technique. Commun. Monogr. 66, 312–324. 10.1080/03637759909376482

[B57] Olivola C. Y. Shafir E. (2013). The martyrdom effect: when pain and effort increase prosocial contributions. J. Behav. Decis. Mak. 26, 91–105. 10.1002/bdm.767 23559692 PMC3613749

[B58] Onwezen M. C. Bartels J. Antonides G. (2014). The self-regulatory function of anticipated pride and guilt in a sustainable and healthy consumption context. Eur. J. Soc. Psychol. 44, 53–68. 10.1002/ejsp.1991

[B59] Rai T. S. Fiske A. P. (2011). Moral psychology is relationship regulation: moral motives for unity, hierarchy, equality, and proportionality. Psychol. Rev. 118, 57–75. 10.1037/a0021867 21244187

[B60] Regan J. W. (1971). Guilt, perceived injustice, and altruistic behavior. J. Pers. Soc. Psychol. 18, 124–132. 10.1037/h0030712 5550433

[B61] Roseman I. J. Spindel M. S. Jose P. E. (1990). Appraisals of emotion-eliciting events: testing a theory of discrete emotions. J. Pers. Soc. Psychol. 59, 899–915. 10.1037/0022-3514.59.5.899

[B62] Rotella K. N. Richeson J. A. (2013). Body of guilt: using embodied cognition to mitigate backlash to reminders of personal and ingroup wrongdoing. J. Exp. Soc. Psychol. 49, 643–650. 10.1016/j.jesp.2013.02.013

[B63] Sachdeva S. Iliev R. Medin D. L. (2009). Sinning saints and saintly sinners: the paradox of moral self-regulation. Psychol. Sci. 20, 523–528. 10.1111/j.1467-9280.2009.02326.x 19320857

[B64] Slovic P. (2007). “If I look at the mass I will never act”: psychic numbing and genocide. Judgm. Decis. Mak. 2, 79–95. Available online at: http://journal.sjdm.org/jdm7303a.pdf

[B65] Smith K. D. Keating J. P. Stotland E. (1989). Altruism reconsidered: the effect of denying feedback on a victim's status to empathic witnesses. J. Pers. Soc. Psychol. 57, 641–650. 10.1037/0022-3514.57.4.641

[B66] Tangney J. P. Tracy J. (2012). Self-conscious emotions, in Handbook of Self and Identity, 2 Edn., eds Leary M. Tangney J. P. (New York, NY: Guilford Press), 446–478.

[B67] Vanberg C. (2008). Why do people keep their promises? An experimental test of two explanations. Econometrica 76, 1467–1480. 10.3982/ECTA7673

[B68] Västfjäll D. Slovic P. Burns W. Erlandsson A. Koppel L. Asutay E.. (2016). The arithmetic of emotion: Integration of incidental and integral affect in judgments and decisions. Front. Psychol. 7:325. 10.3389/fpsyg.2016.00325 27014136 PMC4782160

[B69] Västfjäll D. Slovic P. Mayorga M. (2015). Pseudoinefficacy: negative feelings from children who cannot be helped reduce warm glow for children who can be helped. Front. Psychol. 6:616. 10.3389/fpsyg.2015.00616 26042058 PMC4434905

[B70] Västfjäll D. Slovic P. Mayorga M. Peters E. (2014). Compassion fade: affect and charity are greatest for a single child in need. PLoS ONE 9:e100115. 10.1371/journal.pone.0100115 24940738 PMC4062481

[B71] Wang X. (2011). The role of anticipated guilt in intentions to register as organ donors and to discuss organ donation with family. Health Commun. 26, 683–690. 10.1080/10410236.2011.563350 22126126

[B72] Weinstein N. Ryan R. M. (2010). When helping helps: autonomous motivation for prosocial behavior and its influence on well-being for the helper and recipient. J. Pers. Soc. Psychol. 98, 222–244. 10.1037/a0016984 20085397

[B73] Wilhelm M. O. Bekkers R. (2010). Helping behavior, dispositional empathic concern, and the principle of care. Soc. Psychol. Q. 73, 11–32. 10.1177/0190272510361435

[B74] Zimmermann A. Abrams D. Doosje B. Manstead A. S. R. (2011). Causal and moral responsibility: antecedents and consequences of group-based guilt. Eur. J. Soc. Psychol. 41, 825–839. 10.1002/ejsp.826

[B75] Zoccola P. M. Green M. C. Karoutsos E. Katona S. M. Sabini J. (2011). The embarrassed bystander: embarrassability and the inhibition of helping. Pers. Individ. Dif. 51, 925–929. 10.1016/j.paid.2011.07.026

