# Supplementary material for: Anticipated Guilt for Not Helping and Anticipated Warm Glow for Helping Are Differently Impacted by Personal Responsibility to Help
Source: Front Psychol. 2016 Sep 28;7:1475. doi: 10.3389/fpsyg.2016.01475 (PMC5039200; doi:10.3389/fpsyg.2016.01475)
Supplement: Supplementary file 1 [file Presentation1.PDF]

Appendix 1:

English translations of the included helping scenarios and its respective versions

Study 1a

All participants read the **black** text
Participants reading the high responsibility version of the scenario read the **bold red** text
Participants reading the low responsibility version of the scenario read the ***italics blue*** text

---

Expectation Scenario (also included in Study 2)

Your grandmother has had a good life, but now she is on her death bed. She is physically weak but clear mentally. Your grandmother has always been enthusiastic about different types of volunteering – most notably for the "The City Mission" and you know that this has meant a lot for her during her whole life. **You also know that your grandmother has told other members of the family that she expects you to feel the same kind of enthusiasm for volunteering as she has done and that she would be disappointed if you would not feel it.** [*You also know that your grandmother has told other members of your family that she does not expect you to feel the same kind of enthusiasm for volunteering as she does, but want you to follow your own heart*]. You have a nice last conversation about her life. The following night your grandmother passes away quietly.

The following day, you are going downtown to visit the funeral agency. You suddenly get stopped by a solicitor from "The City Mission". You are asked if you could sign up for volunteering. If you sign your name you will volunteer at a soup kitchen run by the City Mission one day per month.

Effort Scenario (also included in Study 2)

Blood donations and blood transfusions are very direct ways to save a life (you are giving away a part of yourself). In many places there is currently an acute need for blood from certain blood-groups. You know that you have a rare blood-group and that there currently are many patients that need exactly your type of blood.

To give blood does not imply any big effort from you – you only need to visit the blood donor clinic on your way home from work. It would take at most 30 minutes. [*To give blood implies a pretty big effort from you – you would need to take a day off from work, drive 150 kilometers to the nearest blood donor clinic and then 150 kilometers back. It would take at least 4 hours*]

Request Scenario (also included in Study 2)

You have just arrived to your hometown after going with a train that was much delayed due to a storm. All the public transportation has stopped going because it is very late at night. You live close to the train station so you can walk, but for passengers going far, this is not an option. A small man with worn clothes and much packing, seemingly from Thailand and here as a guest worker, asks you in broken English how to get to a certain address. You know that the address is far from the station and that he should take a taxi for around 300SEK ($35). **The man gets obviously anxious and says nervously that he only brought money for the bus-ticket and that he does not have a credit card to pay for a taxi. He also says that it seems very cold for walking. He is close to crying and askes you if you could pay for the taxi. He is not at all aggressive but seems desperate and in distress.** *[The man is surprised to learn this and calmly says that he only brought money for the bus-ticket and that he does not have a credit card to pay for a taxi. He also says that although it is cold he will be probably able to walk there. He thanks you for your time and apologizes for disturbing you.]*

Social closeness Scenario

You are on a city bus on your way to your new job. When you look out the window you see a girl sitting on a bench crying heavily. At appears to you that the girl has been robbed or got something stolen from her. She seems very much in distress but not physically hurt. When the bus makes a turn you see that the crying girl is **your cousin and close friend** *[a girl you recognize from your local supermarket]*. You forgot your cell phone so you cannot call anyone. **Your cousin** *[The girl]* has not seen you.

You can choose to exit the bus on the next stop to quickly get to and try to comfort **your crying cousin** *[the crying girl]*, but doing so would mean that you would be late for work on your first day. You can also remain on the bus to make it on time to work, and hope that someone else helps **your cousin** *[the girl]*.

Bystander Scenario

It is Saturday afternoon and you are currently biking from your home to the liquor-store in order to buy wine for a party this evening. You are running late and hurry in order to arrive before the store closes. When biking through a park you pass a playground. **The playground is totally empty except an 8-year old boy climbing on a jungle gym.** *[One 8-year old boy is climbing the jungle gym but there are many other children and the playground, and a group of parents are having coffee there as well.]* The jungle gym is right next to the biking path. When passing, you see that the boy slips and falls. The boy starts crying heavily and has a nose bleed. He is not seriously injured but the boy cries and bleeds from the nose. **Nobody else has noticed the boy falling.** *[The parents do not initially notice the fall, but it is just a matter of time before they will notice it.]*

You can either stop and help the boy get to his parents (implying that you will not be able to buy the wine), or keep biking in order to make it to the store before it closes.

Study 1b

All participants read the **black** text
Participants reading the high responsibility version of the scenario read the **bold red** text
Participants reading the low responsibility version of the scenario read the ***italics blue*** text

---

Type of Request Scenario
It is Friday afternoon and you are on your way to shop at your local supermarket. You just received your monthly pay and Christmas is approaching. Outside the supermarket you see Bertil. Bertil is 76 years old and well known for you and others in the city. Since his wife died he lives alone in a very old and unmodern house without electricity. Although he has never abused any drug he lives below the poverty limit. He is very shy, but has never bothered anyone and never asked anyone for money. From a distance, you see Bertil about to enter the supermarket and he takes out 5-6 bills from his pocket.

Suddenly he slips and drops all the bills, and before he can pick them up they have blown down a water drain. You see that Bertil gets extremely sad. **For a second you think that you could help Bertil by giving him 50SEK ($6) so he can buy something.** *[For a second you think that you could help Bertil by inviting Bertil home for dinner so he can eat a proper meal].*

Resources Scenario
You have been lucky and just won **50000SEK ($5600)** *[500SEK ($56)]* on a scratch ticker you got as a Christmas gift. Later the same day, you are going downtown for some shopping. You get asked by a very nice and polite solicitor from the Red Cross organization if you would imagine donating a one-time sum of 300SEK ($34) to a fund designated for helping the victims of a recent hurricane in the Philippines. 300SEK is enough for one family to get shelter and clean water for a month. You receive the bank information so you can transfer the money at a later time.

Cause (Money) Scenario (also included in Study 2)
You work at a company with around 100 employees. One of your coworkers (Julia – a single mother in her thirties) accidently forgot her purse in the staff-room purse the other night and unfortunately it got stolen. The thief managed to withdraw 20000SEK ($2240) from her bank account before she noticed anything. **One reason for the theft is that you opened but forgot to close a window in the staff room the evening before and that the thief could enter through the open window.** *[One reason for the theft is that Julia herself opened but forgot to close a window in the staff room the evening before and that the thief could enter through the open window]*

The theft has caused problems for Julia. The insurance money will not be available until later which means that she hardly can buy anything for her or for her children before Christmas. You have some money on your savings account, and for a moment you think that maybe you could pay her liability and lend her 5000SEK ($560) so she can buy Christmas food and presents for her children.

Promise Scenario
A female acquaintance from your high school years are soon about to move to Stockholm. A couple of months ago you met her at a big party. While eating and drinking wine, your acquaintance told you that she worried some about the practical aspects of moving. For example she told you that she was living on the fourth floor in an old building without an elevator, and she was worried about how to get the furniture there without an elevator. **You told her that she should not worry and that you promised her that you would help her move if she needed help. She got very happy for you offering to helping.** *[You told her that you understood her feeling, but that you unfortunately could not help her during that weekend. She said she understood that and that it was all right]*

A couple of days before your acquaintance moving, you see on Facebook that she has created an event where those friends who can help her move can sign up. **You remember your earlier promise at the party, and assume that the acquaintance probably expects you to sign up.** *[You remember that you told her you could not help her, and assume that she probably do not expect you to sign up]*

Cause (Time) Scenario
You are driving your car on the way to the cinema to see a movie premiere that you for a long time have looked forward to see. **When you approach a stoplight you lose your attention for a bit and accidently collide with a car that has stopped for a red light.** *[When you approach a stoplight you are suddenly run into by another car]* In the other car is a middle-aged couple. You get out of your cars and exchange insurance information. You both agree that the accident was **100% your fault.** *[100% their fault.]*

You are all uninjured but the suspension on their car has broken so they are forced to park their car on a nearby parking lot. Your car has only a dent so you can drive on without problems.

You see that the middle-aged couple has briefcases in their car and understand from their conversation that they are on their way to the airport for a vacation. They are trying to call a taxi, but they seem to have a problem with their cell phone. For a minute you think that you could drive the couple to the airport in your car. It is 80kilometers away so this would imply missing the movie premiere.

Study 3 and 4

The alternative endings are here shown in the order they were presented for the participants in Study 3 unless stated otherwise. Note that the order of the alternative endings was reversed in Study 4.

Effort (Study 3 and Study 4)

You walk past the train station a Sunday evening on your way home from a friend. From the main entrance of the station, you see a man walking that seems to come from China. He looks a little lost and tired and he is wearing a big backpack on his back. He approaches you and asks in broken English about an address that you recognize. You know that the address is pretty far away (6km) and that the busses do not go there at this late hour.

Alternative endings

a) *Minimal effort:* Small effort (e.g. explain that it is too far to walk and that he needs a taxi, point at them)
b) *Small effort:* Certain effort (e.g. google the address on your mobile, show and explain the way form where you are now)
c) *Some effort:* Rather a lot of effort (e.g. explain that it is too far to walk, follow him to the taxis and explain to the taxi-driver where he is going)
d) *Big effort:* A lot of effort (e.g. explain that it is too far to walk, follow him to the taxis, explain to the taxi-driver where he is going and pay his taxi-journey of 300SEK)
e) *Extreme effort:* Extreme effort (e.g. explain that it is too far to walk, follow him to the taxis, explain to the taxi-driver where he is going, pay his taxi-journey of 300SEK and go with him in the taxi to the address)

Victim's fault (Study 3)

A classmate of yours has lost her bike. She really needs a bike to get from her home to her lectures and to her work, but at the moment she cannot afford to buy a new one. She asks every one that she knows if they can help. You, and no one else, know that you have a spare bike in your storage room.

Alternative endings

a) *Totally innocent victim*: The friend had done nothing wrong but had merely been unlucky (e.g. she got the bike stolen although it was locked with two locks and standing inside a storage room)
b) *Slightly careless victim*: The friend had done nothing directly wrong but had been a bit careless (e.g. she got her bike stolen when she had parked it unlocked outside a shop for a few minutes)
c) *Careless victim*: The friends had done nothing directly wrong but had been very careless (e.g. she got her bike stolen when it was standing in a bicycle stand in the city centre for a few days)
d) *Somehow victims fault*: The friend had lost her bike due to her own mistake (e.g. she had, during a night out, simply forgotten where she put the bike and never found it)
e) *Clearly victims fault*: The friend had lost her bike all on her own (e.g. she ruined it by throwing it out of a balcony at a party)

Fault (Study 4)

When you leave your apartment to go to school on Monday morning, you see the police in your neighbour’s apartment. You ask what has happened and the neighbour answers that there has been a break-in his apartment last night and that amongst other things his new TV was stolen. When you peak in, you see furniture lying in a mess in the apartment. The police say that it seems like someone entered through the gate of the apartment building during the night and that someone must have missed to close it.

Alternative endings (note that the order of the alternative endings here are shown in the order they were presented in Study 4)

a) *Clearly victims fault*: Indirectly the affected neighbours fault (e.g. your neighbour left the gate open last night to air out)
b) *Maybe the victims fault*: Maybe the affected neighbours fault (e.g. he went shopping last night and think that he forgot to close the gate properly behind him)
c) *Nobody’s fault*: Neither the affected neighbour nor your fault (e.g. he was on a business trip and you were in your apartment all weekend to watch movies)
d) *Maybe your fault*: Maybe your fault (e.g. you left the gate open last night when you went out with the garbage and cannot remember if you closed it)
e) *Clearly your fault*: Your fault due to gross neglect (e.g. you let a shady person into the stairways that you did not recognize last night)

Bystander (Study 3 and Study 4)

You are on your way home to a friend who turns even and who is having a big birthday party. You are wearing your new fancy clothes and are in a hurry as you promised your friend to be there 10 minutes ago to help prepare. On your way to your friend you pass through a park. In the middle of the park you suddenly see a woman falling from her bike and landing in a muddy puddle with the bike next to her. The woman grabs her leg and seems hurt. To try to help the woman would mean that your fancy clothes got dirty.

Alternative endings

a) *No bystanders*: No one else but you can help (only you are nearby)
b) *One bystander*: One other person in a far distance that can help (e.g. someone is jogging on the other side of the park and sees the woman as well)
c) *Some bystanders*: Some other people that can help (e.g. two couples that sit on benches in the middle of the park that see the woman as well)
d) *Several bystanders*: Many other people nearby the woman that can help (e.g. a group of softball-playing students right next to the woman that see her as well)
e) *Many eligible bystanders*: Many other people that can help, and several of them are both closer and more suitable (e.g. a group of polices right next to the woman that see her as well)

Closeness (Both Study 3 and Study 4)

You are on the bus on your way home from school and there are a few stops left before it is your stop. When you look out the window you see a person carrying a big armchair that looks very heavy. The person is staggering ahead and has a forced expression on the face. There is a stop a shortly ahead and someone has already pressed the stop-button to get off.

Alternative endings

a) *Extremely close*: Extremely close to you (e.g. the person is your mother or father)
b) *Very close*: Very close to you (e.g. the person is your friend)
c) *Close*: Close to you (e.g. the person is your classmate)
d) *Somehow distant*: A little close to you (e.g. the person is an acquaintance of a friend and whom you have met once)
e) *Distant*: Not close to you (e.g. the person is someone you have seem in your local grocery shop a few times)
