# Supplementary material for: Anticipated Guilt for Not Helping and Anticipated Warm Glow for Helping Are Differently Impacted by Personal Responsibility to Help
Source: Front Psychol. 2016 Sep 28;7:1475. doi: 10.3389/fpsyg.2016.01475 (PMC5039200; doi:10.3389/fpsyg.2016.01475)
Supplement: Supplementary file 2 [file Presentation2.PDF]

Appendix 2: Additional analyses

1. Pilot study results for Study 1

101 participants read the six vignettes included in Study 1a. Each participant read three of the vignettes in the low-responsibility version and the other three in the high-responsibility version. A high-responsibility version always followed a low-responsibility version and the other way around. After each vignette, they rated the perceived responsibility to help by circling one number between 0 (No responsibility at all) and 10 (Very much responsibility). Table shows means and t-test statistics.

	High-responsibility version	Low responsibility version	t-test statistic
Study 1a			
**Expectation**	5.66 (3.29)	3.98 (2.94)	t(99) = 2.70, p = .008
**Effort**	8.12 (3.13)	5.10 (3.08)	t(99) = 4.89, p < .001
**Request**	5.98 (2.58)	4.24 (2.54)	t(97) = 3.39, p = .001
**Social Closeness**	7.00 (2.77)	4.37 (2.47)	t(97) = 4.99, p < .001
**Bystanders**	7.86 (2.87)	7.42 (3.02)	t(96) = 0.74, p = .458
**Social norm (excluded)**	5.59 (3.38)	5.73 (3.45)	t(98) = -0.21, p = .831
Study 1b			
**Type of helping**	7.58 (3.04)	6.16 (2.76)	t(103) = 2.52, p = .013
**Resources**	5.85 (3.76)	4.48 (2.95)	t(107) = 2.15, p = .034
**Cause [Money]**	7.52 (2.53)	4.67 (2.74)	t(104) = 5.51, p < .001
**Promise**	8.05 (2.64)	2.65 (1.81)	t(106) = 12.13, p < .001
**Cause [Time]**	8.71 (2.41)	4.42 (3.12)	t(103) = 7.94, p < .001
**Success (excluded)**	3.24 (2.79)	3.42 (2.56)	t(106) = -0.34, p = .731

2. Main effects (and interaction effects) for all reported analyses

Study 1a:

If all five vignettes aggregated.
Main effect on version (high/low responsibility): $F(1,169) = 26.63$, $p < .001$, $\eta^2 = .136$
Main effect on type of reaction (anticipated guilt/warm glow): $F\ (1,169) = 54.30$, $p < .001$, $n^2 = .243$
Interaction effect: $F\ (1,169) = 26.91$, $p < .001$, $n^2 = .137$

Expectation vignette.
Main effect on version (high/low responsibility): $F(1,167) = 1.18$, $p =.279$, $\eta^2 = .007$
Main effect on type of reaction (anticipated guilt/warm glow): $F\ (1,167) = 21.06$, $p < .001$, $n^2 = .112$
Interaction effect: $F\ (1,167) = 5.11$, $p = .025$, $n^2 = .030$

Effort vignette.
Main effect on version (high/low responsibility): $F(1,167) = 0.98$, $p =.323$, $\eta^2 = .006$
Main effect on type of reaction (anticipated guilt/warm glow): $F\ (1,167) = 65.76$, $p < .001$, $n^2 = .283$
Interaction effect: $F\ (1,167) = 8.59$, $p = .004$, $n^2 = .049$

Request vignette.
Main effect on version (high/low responsibility): $F(1,167) = 3.40$, $p =.067$, $\eta^2 = .020$
Main effect on type of reaction (anticipated guilt/warm glow): $F\ (1,167) = 10.49$, $p = .001$, $n^2 = .059$
Interaction effect: $F(1,167) = 3.40$, $p =.067$, $\eta^2 = .020$

Closeness vignette.
Main effect on version (high/low responsibility): $F(1,167) = 18.87$, $p <.001$, $\eta^2 = .102$
Main effect on type of reaction (anticipated guilt/warm glow): $F\ (1,167) = 6.39$, $p = .012$, $n^2 = .037$
Interaction effect: $F(1,167) = 8.54$, $p =.004$, $\eta^2 = .049$

Bystander vignette.
Main effect on version (high/low responsibility): $F(1,167) = 14.11$, $p <.001$, $\eta^2 = .078$
Main effect on type of reaction (anticipated guilt/warm glow): $F\ (1,167) = 5.68$, $p = .018$, $n^2 = .033$
Interaction effect: $F(1,167) = 5.74$, $p =.018$, $\eta^2 = .033$

Study 1b:

If all five vignettes aggregated.
Main effect on version (high/low responsibility): $F(1,168) = 63.25$, $p < .001$, $\eta^2 = .274$
Main effect on type of reaction (anticipated guilt/warm glow): $F(1,168) = 22.79$, $p < .001$, $n^2 = .119$
Interaction effect: $F(1,168) = 50.65$, $p < .001$, $n^2 = .232$

Type of helping vignette.
Main effect on version (high/low responsibility): $F(1,166) = 8.02$, $p =.005$, $\eta^2 = .046$
Main effect on type of reaction (anticipated guilt/warm glow): $F(1,166) = 23.39$, $p < .001$, $n^2 = .123$
Interaction effect: $F(1,166) = 4.35$, $p = .039$, $n^2 = .026$

Resources vignette.
Main effect on version (high/low responsibility): $F(1,166) = 2.29$, $p =.132$, $\eta^2 = .014$
Main effect on type of reaction (anticipated guilt/warm glow): $F(1,166) = 8.58$, $p = .004$, $n^2 = .049$
Interaction effect: $F(1,166) = 3.47$, $p = .064$, $n^2 = .020$

Cause (Money) vignette.
Main effect on version (high/low responsibility): $F(1,166) = 2.69$, $p =.103$, $\eta^2 = .016$
Main effect on type of reaction (anticipated guilt/warm glow): $F(1,166) = 7.28$, $p = .008$, $n^2 = .042$
Interaction effect: $F(1,166) = 10.10$, $p =.002$, $\eta^2 = .057$

Promise vignette.
Main effect on version (high/low responsibility): $F(1,166) = 38.05$, $p <.001$, $\eta^2 = .186$
Main effect on type of reaction (anticipated guilt/warm glow): $F(1,166) = 24.23$, $p < .001$, $n^2 = .127$
Interaction effect: $F(1,166) = 25.77$, $p <.001$, $\eta^2 = .134$

Cause (Time) vignette.
Main effect on version (high/low responsibility): $F(1,166) = 10.39$, $p =.002$, $\eta^2 = .059$
Main effect on type of reaction (anticipated guilt/warm glow): $F(1,166) = 4.86$, $p = .029$, $n^2 = .028$
Interaction effect: $F(1,166) = 5.12$, $p =.025$, $\eta^2 = .030$

If three vignettes aggregated (not including the cause-vignettes).
Main effect on version (high/low responsibility): $F(1,168) = 34.18$, $p < .001$, $\eta^2 = .169$
Main effect on type of reaction (anticipated guilt/warm glow): $F(1,168) = 28.76$, $p < .001$, $n^2 = .146$
Interaction effect: $F(1,168) = 30.40$, $p < .001$, $n^2 = .153$

Anticipated guilt if not helping in high responsibility versions 3.55 (1.52) and in low responsibility versions 2.23 (1.42), $t(87) = 7.42$, $p < .001$. Anticipated warm glow if helping in high responsibility versions 3.85 (1.25) and in low responsibility versions 3.81 (1.25), $t(81) = 0.26$, $p = .793$.

Study 2

If all four vignettes aggregated

Main effect on version (high/low responsibility): $F(1,190) = 15.28$, $p < .001$, $\eta^2 = .074$
Main effect on type of reaction (anticipated guilt/warm glow): $F\ (1,190) = 173.19$, $p < .001$, $n^2 = .477$
Interaction effect: $F\ (1,190) = 57.49$, $p < .001$, $n^2 = .232$

Expectation vignette.
Main effect on version (high/low responsibility): $F(1,188) = 2.09$, $p =.150$, $\eta^2 = .011$
Main effect on type of reaction (anticipated guilt/warm glow): $F\ (1,188) = 89.43$, $p < .001$, $n^2 = .322$
Interaction effect: $F\ (1,188) = 7.28$, $p = .008$, $n^2 = .037$

Effort vignette.
Main effect on version (high/low responsibility): $F(1,187) = 0.09$, $p =.771$, $\eta^2 < .001$
Main effect on type of reaction (anticipated guilt/warm glow): $F\ (1,187) = 189.77$, $p < .001$, $n^2 = .504$
Interaction effect: $F\ (1,187) = 11.28$, $p = .001$, $n^2 = .057$

Cause vignette.
Main effect on version (high/low responsibility): $F(1,186) = 4.17$, $p =.043$, $\eta^2 = .022$
Main effect on type of reaction (anticipated guilt/warm glow): $F\ (1,186) = 18.89$, $p < .001$, $n^2 = .092$
Interaction effect: $F\ (1,186) = 40.04$, $p = .001$, $n^2 = .177$

Request vignette.
Main effect on version (high/low responsibility): $F(1,186) = 3.49$, $p =.063$, $\eta^2 = .018$
Main effect on type of reaction (anticipated guilt/warm glow): $F\ (1,186) = 89.74$, $p < .001$, $n^2 = .325$
Interaction effect: $F\ (1,186) = 3.96$, $p = .048$, $n^2 = .021$

If three vignettes aggregated (not including the cause vignette)

Main effect on version (high/low responsibility): $F(1,190) = 7.95$, $p = .005$, $\eta^2 = .040$
Main effect on type of reaction (anticipated guilt/warm glow): $F\ (1,190) = 197.82$, $p < .001$, $n^2 = .510$
Interaction effect: $F\ (1,190) = 24.49$, $p < .001$, $n^2 = .114$

Anticipated guilt if not helping in high responsibility versions 3.86 (1.57) and in low responsibility versions 3.28 (1.61), $t(191) = 4.99$, $p < .001$. Anticipated warm glow if helping in high responsibility versions 4.90 (1.39) and in low responsibility versions 5.00 (1.44), $t(191) = -1.00$, $p = .319$.

Study 3

Effort scenario

Main linear effect on version (high → low responsibility): $F(1,103) = 26.25$, $p < .001$, $\eta^2 = .203$
Main effect on type of reaction (anticipated guilt/warm glow): $F(1,103) = 24.19$, $p < .001$, $n^2 = .190$
Interaction effect: $F(1,103) = 162.43$, $p < .001$, $n^2 = .612$

Victim's fault scenario

Main linear effect on version (high → low responsibility): $F(1,103) = 427.60$, $p < .001$, $\eta^2 = .806$
Main effect on type of reaction (anticipated guilt/warm glow): $F(1,103) = 4.02$, $p = .048$, $n^2 = .038$
Interaction effect: $F(1,103) = 3.47$, $p = .065$, $n^2 = .033$

Bystander scenario

Main linear effect on version (high → low responsibility): $F(1,103) = 164.37$, $p < .001$, $\eta^2 = .615$
Main effect on type of reaction (anticipated guilt/warm glow): $F(1,103) = 1.26$, $p = .264$, $n^2 = .012$
Interaction effect: $F(1,103) = 109.64$, $p <.001$, $n^2 = .516$

Closeness scenario

Main linear effect on version (high → low responsibility): $F(1,103) = 69.31$, $p < .001$, $\eta^2 = .402$
Main effect on type of reaction (anticipated guilt/warm glow): $F(1,103) = 9.60$, $p = .003$, $n^2 = .085$
Interaction effect: $F(1,103) = 114.71$, $p <.001$, $n^2 = .527$

Study 4

Effort scenario

Main linear effect on version (low → high responsibility): $F(1,109) = 42.43$, $p < .001$, $\eta^2 = .280$
Main effect on type of reaction (anticipated guilt/warm glow): $F(1,109) = 105.12$, $p < .001$, $n^2 = .491$
Interaction effect: $F(1,109) = 316.69$, $p < .001$, $n^2 = .744$

Fault scenario

Main linear effect on version (low → high responsibility): $F(1,105) = 93.44$, $p < .001$, $\eta^2 = .471$
Main effect on type of reaction (anticipated guilt/warm glow): $F(1,105) = 4.05$, $p = .047$, $n^2 = .037$
Interaction effect: $F(1,105) = 216.06$, $p < .001$, $n^2 = .673$

Bystander scenario

Main linear effect on version (low → high responsibility): $F(1,109) = 319.12$, $p < .001$, $\eta^2 = .745$
Main effect on type of reaction (anticipated guilt/warm glow): $F(1,109) = 38.78$, $p < .001$, $n^2 = .262$
Interaction effect: $F(1,109) = 157.54$, $p < .001$, $n^2 = .591$

Closeness scenario

Main linear effect on version (low → high responsibility): $F(1,107) = 191.69$, $p < .001$, $\eta^2 = .642$
Main effect on type of reaction (anticipated guilt/warm glow): $F(1,107) = 39.35$, $p < .001$, $n^2 = .269$
Interaction effect: $F(1,107) = 315.79$, $p < .001$, $n^2 = .747$

3. Anticipated guilt and anticipated warm glow and its relation to helping intentions

Study 3 and Study 4 included a single-item question about intention to help in each version of each helping scenario. Because we focus on how different anticipated emotions are influenced by degree of perceived responsibility, we have not included these analyses in the manuscript. As we realize that this information could be on interest for readers, we wish to disclose the findings related to the helping intention variable as well. The results from further analyses as well as the raw data and statistical code can be provided upon request.

In Study 3, participants first read all versions of all helping scenarios and rated either their anticipated guilt if not helping or their anticipated warm glow if helping. Then they read the same versions of the same helping scenarios again and this time the rated how probably it was that they would help in each version. Answering was done on a scale ranging from 0 = not probable at all to 10 = very probable.

Mean helping intention in each alternative ending in Study 3. Standard deviation in parenthesis

Alt. Ending	Effort	Victim fault	Bystanders	Closeness
A (high res)	9.16 (1.67)	9.21 (1.56)	9.13 (1.45)	9.11 (1.83)
B	8.50 (2.03)	8.26 (2.14)	8.39 (1.83)	8.90 (1.65)
C	6.71 (2.80)	6.18 (3.11)	7.48 (2.19)	6.68 (2.38)
D	2.13 (2.54)	4.98 (3.32)	6.29 (2.76)	3.79 (2.80)
E (low res)	1.26 (2.32)	2.56 (3.25)	4.07 (3.32)	2.47 (2.92)

Mean helping intention in each alternative ending in Study 3 as a function of if participants first imagined not helping (anticipated guilt) or first imagined helping (anticipated warm glow). Standard deviation in parenthesis

	Effort		Victim fault		Bystanders		Closeness	
Alt. Ending	Guilt	Warm glow	Guilt	Warm glow	Guilt	Warm glow	Guilt	Warm glow
A (high res)	9.14 (1.69)	9.18 (1.72)	9.32 (1.32)	9.11 (1.76)	9.12 (1.45)	9.13 (1.47)	9.40 (1.09)	8.85 (2.28)
B	8.40 (2.19)	8.60 (1.89)	8.56 (2.10	7.98 (2.16)	8.43 (1.76)	8.36 (1.91)	9.16 (1.22)	8.65 (1.95)
C	6.38 (2.94)	7.02 (2.66)	6.72 (3.27)	5.69 (2.89)	7.14 (2.09)	7.78 (2.26)	6.38 (2.34)	6.95 (2.41)
D	1.44 (2.16)	2.76 (2.71)	5.42 (3.42)	4.58 (5.42)	5.57 (2.85)	6.93 (2.54)	2.98 (2.62)	4.53 (2.77)
E (low res)	0.66 (1.77)	1.80 (2.63)	2.80 (3.28)	2.35 (3.23)	2.73 (2.67)	5.25 (3.41)	1.36 (2.28)	3.47 (3.08)

Correlation coefficients showing the bivariate relations between anticipated guilt and helping intention and between anticipated warm glow and helping intention in each alternative ending in Study 3.

	Effort		Victim fault		Bystanders		Closeness	
Alt. Ending	Guilt	Warm glow	Guilt	Warm glow	Guilt	Warm glow	Guilt	Warm glow
A (high res)	.39	-.01	.55	.17	.48	.26	.70	.33
B	.49	.08	.62	.20	.59	.14	.68	.19
C	.53	.01	.77	.27	.46	.07	.42	.18
D	.44	.03	.81	.36	.59	.11	.59	.13
E (low res)	.32	.14	.74	.44	.64	.42	.77	.13

In Study 4, participants read all versions of all helping scenarios and rated both their anticipated guilt if not helping, their anticipated warm glow if helping, and lastly how probable it was that they would help for each version.

Mean helping intention in each version (alternative ending) in Study 4. Standard deviation in parenthesis.

	Effort	Fault	Bystanders	Closeness
Alt. Ending				
A (low res)	1.73 (1.48)	3.93 (2.56)	3.60 (2.54)	2.37 (2.18)
B	2.12 (1.78)	4.29 (2.48)	4.73 (2.51)	3.53 (2.38)
C	6.70 (2.40)	4.82 (2.53)	5.90 (2.40)	5.50 (2.55)
D	7.41 (2.55)	6.70 (2.44)	7.33 (2.32)	7.79 (2.31)
E (high res)	8.18 (2.46)	8.06 (2.30)	9.02 (1.60)	9.14 (1.63)

Note: Please note that the order of the alternative endings is different in Study 3 and Study 4.

Correlation coefficients showing the bivariate relations between anticipated guilt and helping intention and between anticipated warm glow and helping intention in each version (alternative ending) in Study 4.

	Effort		Fault		Bystanders		Closeness	
Alt. Ending	Guilt	Warm glow	Guilt	Warm glow	Guilt	Warm glow	Guilt	Warm glow
A (low res)	.50	.19	.61	.36	.75	.45	.69	.22
B	.62	.15	.66	.41	.84	.41	.78	.17
C	.75	.25	.74	.39	.80	.53	.83	.05
D	.59	.23	.79	.28	.83	.55	.79	-.00
E (high res)	.51	.26	.81	-.01	.74	.36	.73	-.03
